# Gene expression profiles of immune-regulatory genes in whole blood of cattle with a subclinical infection of *Mycobacterium avium* subsp. *paratuberculosis*

**DOI:** 10.1371/journal.pone.0196502

**Published:** 2018-04-26

**Authors:** Hyun-Eui Park, Hong-Tae Park, Young Hoon Jung, Han Sang Yoo

**Affiliations:** 1 Department of Infectious Disease, College of Veterinary Medicine, Seoul National University, Seoul, Republic of Korea; 2 National Institute of Animal Science, Rural Development Administration, Wanju, Republic of Korea; 3 Institute of Green Bio Science and Technology, Seoul National University, Pyeongchang, Republic of Korea; Universita degli Studi di Sassari, ITALY

## Abstract

Johne’s disease is a chronic wasting disease of ruminants caused by *Mycobacterium avium* subsp. *paratuberculosis* (MAP), resulting in inflammation of intestines and persistent diarrhea. The initial host response against MAP infections is mainly regulated by the Th1 response, which is characterized by the production of IFN-γ. With the progression of disease, MAP can survive in the host through the evasion of the host’s immune response by manipulating the host immune response. However, the host response during subclinical phases has not been fully understood. Immune regulatory genes, including Th17-derived cytokines, interferon regulatory factors, and calcium signaling-associated genes, are hypothesized to play an important role during subclinical phases of Johne’s disease. Therefore, the present study was conducted to analyze the expression profiles of immune regulatory genes during MAP infection in whole blood. Different expression patterns of genes were identified depending on the infection stages. Downregulation of IL-17A, IL-17F, IL-22, IL-26, HMGB1, and IRF4 and upregulation of PIP5K1C indicate suppression of the Th1 response due to MAP infection and loss of granuloma integrity. In addition, increased expression of IRF5 and IRF7 suggest activation of IFN-α/β signaling during subclinical stages, which induced indoleamine 2,3-dioxygenase mediated depletion of tryptophan metabolism. Increased expression of CORO1A indicate modulation of calcium signaling, which enhanced the survival of MAP. Taken together, distinct host gene expression induced by MAP infection indicates enhanced survival of MAP during subclinical stages.

## Introduction

Paratuberculosis (PTB) or Johne’s disease (JD) is a chronic infectious disease leading to persistent diarrhea, progressive wasting, and cachexia, all of which are caused by *Mycobacterium avium* subsp. *paratuberculosis* (MAP) [1]. MAP can affect a range of ruminants, including cattle, goats, lamb, and deer [1] as well as non-ruminants such as parrots, baboons, tamarins, cavies, lemurs, and wallabies [2]. The transmission of PTB usually occurs through the ingestion of contaminated materials such as feed, colostrum, water, and soil [3, 4].

After ingesting contaminated materials, intestinal M cells, which are located in Peyer’s patches in the ileum, uptake and transfer MAP to macrophages that are distributed in the mesenteric lymph nodes [5]. Generally, ingested pathogens are eliminated within the macrophage. However, MAPs can survive in host macrophages by interfering with phagosome maturation [6]. Previous studies have suggested that MAP can inhibit host Rab proteins, which are essential for the phagosome–lysosome fusion following phagocytosis in human and mouse macrophages [7].

Due to the persistence of MAPs, macrophages form granulomas during subclinical phases of infection in the intestinal lymphoid tissue [8]. Previous studies have suggested immunological changes related to fecal shedding during the progression of disease [9, 10]. Fecal shedding has been shown to occur with the downregulation of cell-mediated immune response and upregulation of the humoral immune response *in vivo* [10]. In contrast, some infected animals show increased fecal shedding with the activation of cellular immunity [9]. However, specific mechanisms of the immune response that induces fecal shedding are not clear.

In the subclinical stages, infected macrophages with MAP upregulate expression of CD29, CD56, IL-1α, and TRAF1, resulting in the recruitment of immune cells to the sites of infection [11]. Activated macrophages with MAP move to the local lymph nodes and present an antigen for stimulating naive T cells to induce the Th1 response, which involves the production of interferon gamma and pro-inflammatory cytokines such as IL-6, IL-1α, and IL-2 [12]. Dominance of the Th1 response continue during the subclinical stages, and activated Th1 lymphocytes induce the cell-mediated immune response by producing IL-2, TNF-β, and IFN-γ [12].

During the late subclinical stages of PTB, the Th1 response is gradually diminished and the Th2 response is enhanced, which induces the humoral immune response [13]. With the progression of disease from the subclinical phase to the clinical phase, the cell-mediated immune response totally diminishes, and the humoral immune response, which is characterized by the production of IL-4 and IL-10, becomes prominent [13]. Furthermore, with the shift from the Th1 to Th2 response, clinical signs and lesions become more severe. Therefore, understanding the host response during subclinical phases is critical in identifying the pathogenesis of JD. We hypothesized that immune regulatory genes play an important role in the subclinical phases of JD during the immunological shift from the Th1 to Th2 response.

The Th17-derived cytokine is known to protect hosts from extracellular bacteria causing respiratory and intestinal tract infections [14]. However, several studies have suggested that Th17-derived cytokines may have immune regulatory roles against the infection of intracellular bacteria such as *Salmonella* and *Listeria monocytogenes* [15, 16]. IL-17 deficient mice show enhanced bacterial numbers in the spleen and liver after an infection by *Salmonella enterica* [15]. In addition, with the infection of *Listeria monocytogenes*, bacterial numbers and granuloma formation were increased in the liver [16]. Interferon regulatory factor (IRF) is a transcription factor that has regulatory roles in the immune system [17]. In particular, IRFs regulate the innate immune response via pattern recognition receptor signaling such as TLRs, CLRs, RLRs, and NLRs [18]. High mobility group box 1 (HMGB1) is a non-histone nuclear protein that is involved in the regulation of the immune response [19, 20]. PIP5K1C has been known to have an immune-regulatory function via the modulation of neutrophil polarization and infiltration [21]. In addition, CORO1A have diverse functions, including calcium homeostasis, cytoskeletal dynamics, and maintenance of immune cell diversity and function [22]. However, the role of these immune-regulatory genes in JD has not been yet fully understood. Therefore, we analyzed the expression of immune regulatory genes among cattle groups that have different levels of antibodies to MAP and fecal shedding in order to determine the role of immune-regulatory genes during subclinical phases of JD.

## Materials and methods

### Animals

The animals were selected from a national farm in the mid-west region of South Korea. In this farm, the presence of infectious diseases, including paratuberculosis, bovine tuberculosis, brucellosis, infectious bovine rhinotracheitis, and bovine viral diarrhea, were investigated two times per year in spring and autumn. In total, 79 Holstein cows were selected for the experiments, according to the results of ELISA performed using a commercial ELISA kit (IDEXX Laboratories, Inc., Westbrook, ME, USA) and fecal detection of MAP by PCR. In brief, fecal DNA was extracted using the mGITC/SC method [23] and amplification of IS900 and ISMAP02-targeted real-time PCR was conducted as previously described with slight modification. [24, 25]. In total of 20μl of reaction mixture consisted with 10μl of 2 × Rotor-Gene Probe PCR master mix (Qiagen, Hilden, Germany), 500 nM primers, 200 nM probes, 4 μl fecal DNA, and 4 μl nuclease free water. Real-time PCR reaction was performed under the following conditions: 1 cycle at 95°C for 5 min, followed by 45 cycles at 95#x00B0;C for 15 s, and 60°C for 1 min. The fecal sample was regarded to be positive when both *IS900* and *ISMAP*02 real-time PCR results were positive. Primers used in real-time PCR were listed in Table 1. For the selection of animals, ELISA and fecal PCR were performed 4 times within a 6-month interval to ensure precise classification of animals. For evaluation of clinical status of animals, all animals were monitored for two years for the presence of chronic diarrhea and cachexia. This study was carried out in strict accordance with the guidelines of the Institutional Animal Use and Care Committee of the National Institute of Animal Science. The protocol was approved by the Institutional Animal Use and Care Committee of the National Institute of Animal Science (Permit number 2013–046).

**Table 1:** Oligonucleotide sequences of primers used for real-time PCR.

Target gene		Primer sequence (5′→3′)	PCR product size (base pair)	Reference
Beta actin	F	GCA AGC AGG AGT ACG ATG AG	134	[26]
	R	GCC ATG CCA ATC TCA TCT CG		
IL-17A	F	CAC AGC ATG TGA GGG TCA AC	101	In this study
	R	GTG GAG AGT CCA AGG TGA GG		
IL-17F	F	GAG GAA GCA AAA CGG CTG TC	115	In this study
	R	CTG ATC TGC CAT CGG GTC AT		
IL-22	F	CTG TAG GCT CAA CGA GTC CG	150	In this study
	R	CGC TTC GTC ACC TGA TGG AT		
IL-26	F	AAC GAT TCC AGA AGA TCG CA	164	In this study
	R	CCA CAA AGT GCA TTT CCT TGC		
HMGB1	F	CGA ACA TCC TGG CCT GTC TA	150	In this study
	R	TTA GCT CGG TAT GCG GCA AT		
CORO1A	F	ACC CTG ACA CCA ACA TCG TC	166	In this study
	R	TTG TTC ACC TCC AGA CCA CG		
PIP5K1C	F	GAG ATT GTG GTC CCC AAG GA	191	In this study
	R	CTC CTC TCA TCG GTG GGA AA		
IRF3	F	GAA CCC AAA AGC CTC GGA TAC	162	In this study
	R	CCT GGA AGA TGC CGA AAT CC		
IRF4	F	GCA GAG ATC CCG TAC CAG TG	167	In this study
	R	TCG GCA GAC CTT ATG CTT GG		
IRF5	F	AGA CCT CAA AGA CCG CAT GG	154	In this study
	R	TTA CTG CAT GCC AAC TGG GT		
IRF7	F	CGC AAC GCT TTG TGA TGT TG	146	In this study
	R	TGC AGG TGG GGC ATC TTC TA		
IS900	F	ATG ACG GTT ACG GAG GTG GTT	76	[24]
	R	TGC AGT AAT GGT CGG CCT TAC		
	Probe	FAM-CGA CCA CGC CCG CCC AGA-TAMRA		
ISMAP02	F	CGG CTG GAC ACG GAA TG	67	[25]
	R	CAT GAG CGA CAG TAT CTT TCG AA		
	Probe	JOE-ATC CGT CCC AGT GGC GGA GTC AC-BHQ-1		

### Sampling and extraction of total RNA from whole blood

Peripheral blood samples (3 ml) were collected from the tail vein of cattle with the BD Vacutainer® Plus Plastic K_2_EDTA Tubes and BD Vacutainer® Plus Plastic Serum Tubes. The extraction of total RNA from whole blood was performed as previously described [26]. In brief, 125 μl of whole blood was mixed with the same volume of RNase-free water and 750 μl of Trizol LS reagent (Ambion) and incubated at room temperature for 5 min. Thereafter, 200 μl of chloroform (Sigma-Aldrich) was mixed and centrifuged at 13,523 ×g and 4°C for 15 min. The supernatant was collected into a 1.5 ml tube, mixed with the same volume of 70% ethanol, and then transferred to an RNAeasy column (Qiagen, Hilden, Germany) and centrifuged at 8,500 ×g for 15 sec. After the wash steps, 30 μl of RNase-free water was added and centrifuged at 8,500 ×g for 1 min. Eluted RNA was stored at -80°C until use. For the separation of serum, 3 ml of blood samples were centrifuged at 1,500 ×g for 10 min. Separated serum was transferred to 1.5 ml tube and analyzed for the presence of MAP-specific antibodies using a commercial ELISA kit.

### Selection of immune regulatory genes

Eleven genes that are related to immune regulatory function were selected based on previous studies [27, 28] and classified to three categories as follows; Th17-derived cytokines (IL-17A, IL-17F, IL-22, and IL-26), calcium signaling (HMGB1, CORO1A, and PIP5K1C), and interferon regulatory factors (IRF3, IRF4, IRF5, and IRF7).

### Optimization of real-time PCR conditions

Real-time PCR conditions were optimized with an identical cDNA template for each gene. Five concentrations of both forward and reverse primers ranging from 0.25 μM to 1.25 μM, with a 0.25 μM interval, were tested. In addition, five annealing temperatures from 56°C to 64°C with a two-degree interval were tested. For further experiments, optimal primer concentrations and annealing temperatures that showed the highest fluorescence value were selected for further analysis.

### Real-time PCR

The cDNA was synthesized with random primers using a QuantiTect^®^ Reverse Transcription Kit (Qiagen Inc., Valencia, CA, USA) according to the manufacturer’s instructions. The expression of eleven immune regulatory genes was identified by quantitative real-time RT-PCR with a Rotor-Gene multiplex PCR kit (Qiagen Inc). Briefly, a total of 18 μl reaction mixture included 10 μl of SYBR master mix, RNase-free water, and 0.5 μM forward and reverse primers. Finally, 2 μl of cDNA template was added to the mixture to a final volume of 20 μl. Specific amplification with primers for each target was identified by a homology search (https://www.ncbi.nlm.nih.gov/tools/primer-blast) and agarose gel electrophoresis. The primers used in this study are shown in Table 1. Real-time PCR was performed with triplicate samples at 95°C for 10 min, followed by 45 cycles of 95°C for 15 s and 60°C for 45 s. A no-template sample was used for the negative control. The gene expression was calculated by the 2^-ΔΔ^Ct method with β-actin for the housekeeping gene.

### Statistical analysis

Statistical significance was confirmed by ANOVA with Tukey’s post hoc test among the experimental groups using the GraphPad Prism software version 7.00 (GraphPad Software, Inc., La Jolla, CA, USA). A *P* value of less than 0.05 (*p* < 0.05) was considered as statistically significant, and all experiments were recorded as the means of biological triplicates.

## Results

### Animals

**Fig 1:**
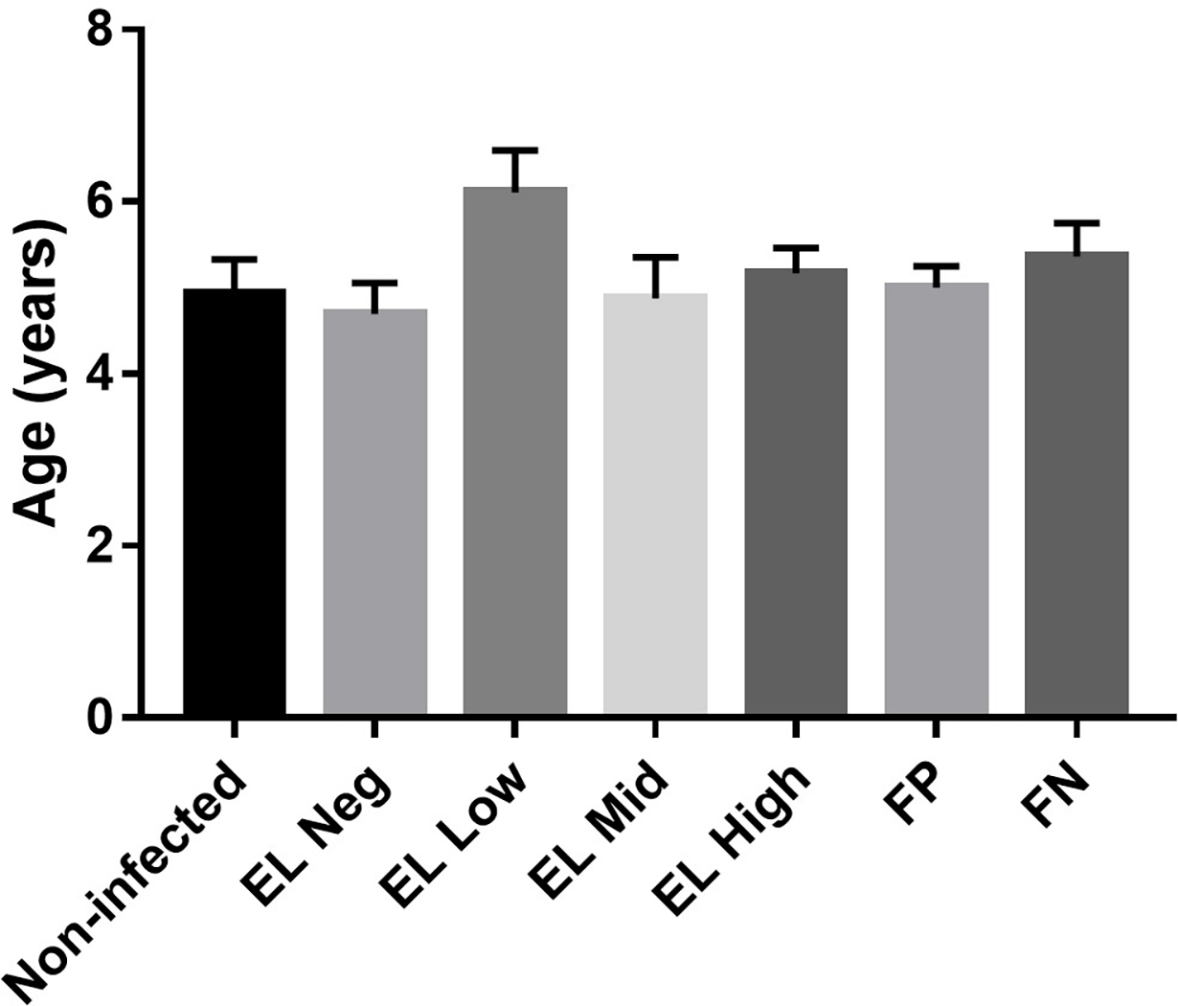
Comparison of mean age among the experimental animals. The mean age of the experimental animals among the experimental groups was represented with a bar graph.

The study subject included 79 heifers that were classified into five groups based on the results of the PCR and ELISA. Non-infected group (n = 27) was defined as those that were ELISA- and PCR-negative during the entire examination. Infected animals were classified into four groups according to the ELISA sample to positive (S/P) ratio. The EL Neg group (n = 23) was defined as those with a S/P ratio < 45 and PCR-positive. The EL Low group (n = 9) was defined as those with a S/P ratio <100 and ≥45. The EL Mid group (n = 8) was defined as those with a S/P ratio <150 and ≥100. The EL High group (n = 12) was defined as those with S/P ratio ≥150. All animals did not show chronic diarrhea and cachexia for two years. Furthermore, infected animals were classified into two groups based on the presence of fecal shedding. The FP group (n = 41) was defined as fecal PCR-positive, and the FN group (n = 11) was defined as fecal PCR- negative. The age of animals ranged from 2 to 10 years, and the mean **±** SD for the age of the animals for each group was as follows: Non-infected group, 4.92 ± 2.09 years; EL Neg group, 4.69 ± 1.74 years; EL Low group 6.11 ± 1.45 years; EL Mid group 4.87 ± 1.35 years; EL High group 5.16 ± 1.02 years; FP group, 5 year ± 1.61 years; FN group, 5.36 ± 1.28 years. Among these groups, age did not show any significant differences (p > 0.05) (Fig 1). Detailed information of all animals was listed in the supplementary materials (S1 Table).

### Optimization of real-time PCR conditions

The optimal concentration of primers and annealing temperature was determined by conducting real-time PCR with three primer concentrations and annealing temperatures. The combination of forward and reverse primers at 0.5 μM and an annealing temperature of 60°C showed the highest florescence and lowest C_T_ value. The combination of 0.5 μM forward and reverse primers and an annealing temperature of 60°C were used in further analyses.

### Gene expression profiles between the infected groups

**Fig 2:**
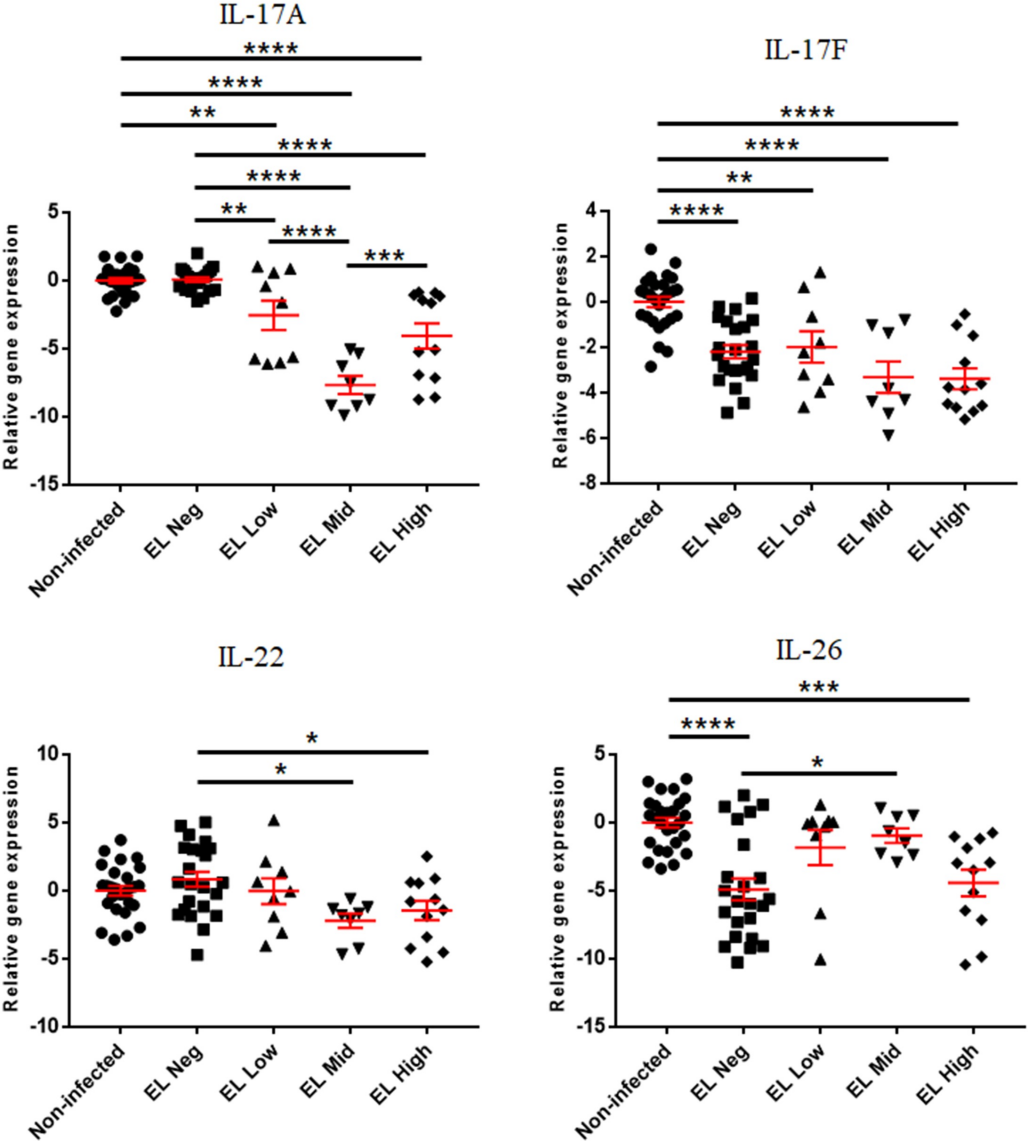
Differences in gene expression levels of Th17-derived cytokine genes between the non-infected, EL Neg, EL Low, EL Mid, and EL High groups. Scatter plots for each gene represent for each individual animal. Values of relative gene expression were normalized to the reference gene β-actin. * indicates a *p*-value <0.05; ** indicates a *p*-value <0.01; *** indicates a *p*-value <0.001; **** indicates a *p*-value <0.0001.

**Fig 3:**
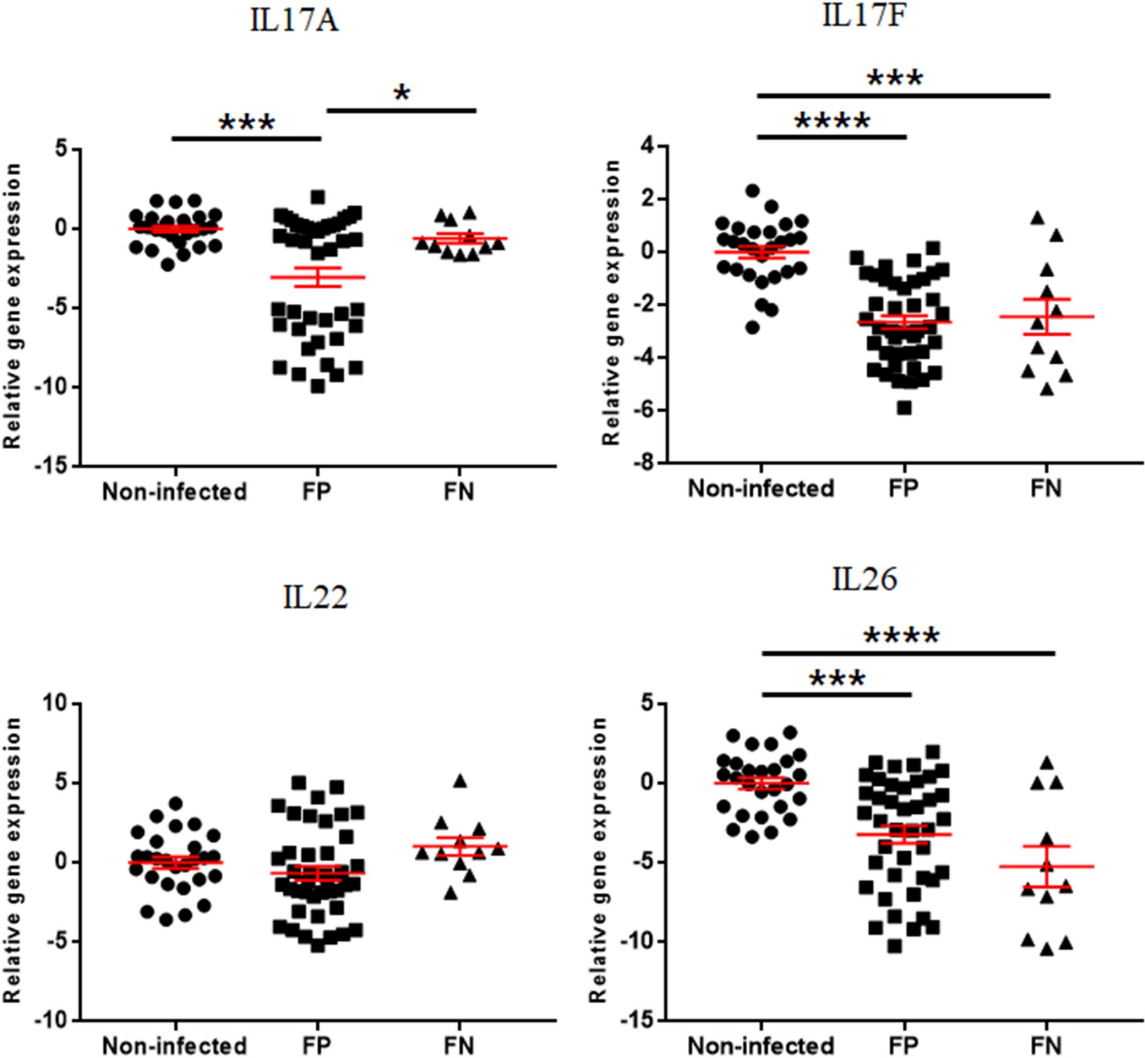
Differences in gene expression levels of Th17-derived cytokine genes between the non-infected, FP, and FN groups. Scatter plots for each gene represent for each individual animal. Values of relative gene expression were normalized to the reference gene β-actin. * indicates a *p*-value <0.05; ** indicates a *p*-value <0.01; *** indicates a *p*-value <0.001; **** indicates a *p*-value <0.0001.

The expression of Th17-derived cytokine genes is presented in Figs 2 and 3. IL-17A was downregulated in the EL Low, Mid, and High groups compared to the non-infected and EL Neg groups. In addition, IL-17A was downregulated in the EL Mid group compared to the EL Low group and upregulated in EL High group compared to the EL Mid group. IL-22 was downregulated in the EL Middle and High groups compared to the EL Neg group. IL-26 was downregulated in EL Neg and High groups compared to the non-infected group. Furthermore, IL-26 was upregulated in the EL Mid group compared to the EL Neg group (Fig 1). When compared with the presence of fecal shedding, 3 genes (IL-17A, IL-17F, and IL-26) were downregulated in the FP group compared to the non-infected group. In addition, two genes (IL-17F and IL-26) were downregulated in the FN group compared to the non-infected group. Furthermore, the expression of IL-17A was increased in the FN group compared to the FP group.

**Fig 4:**
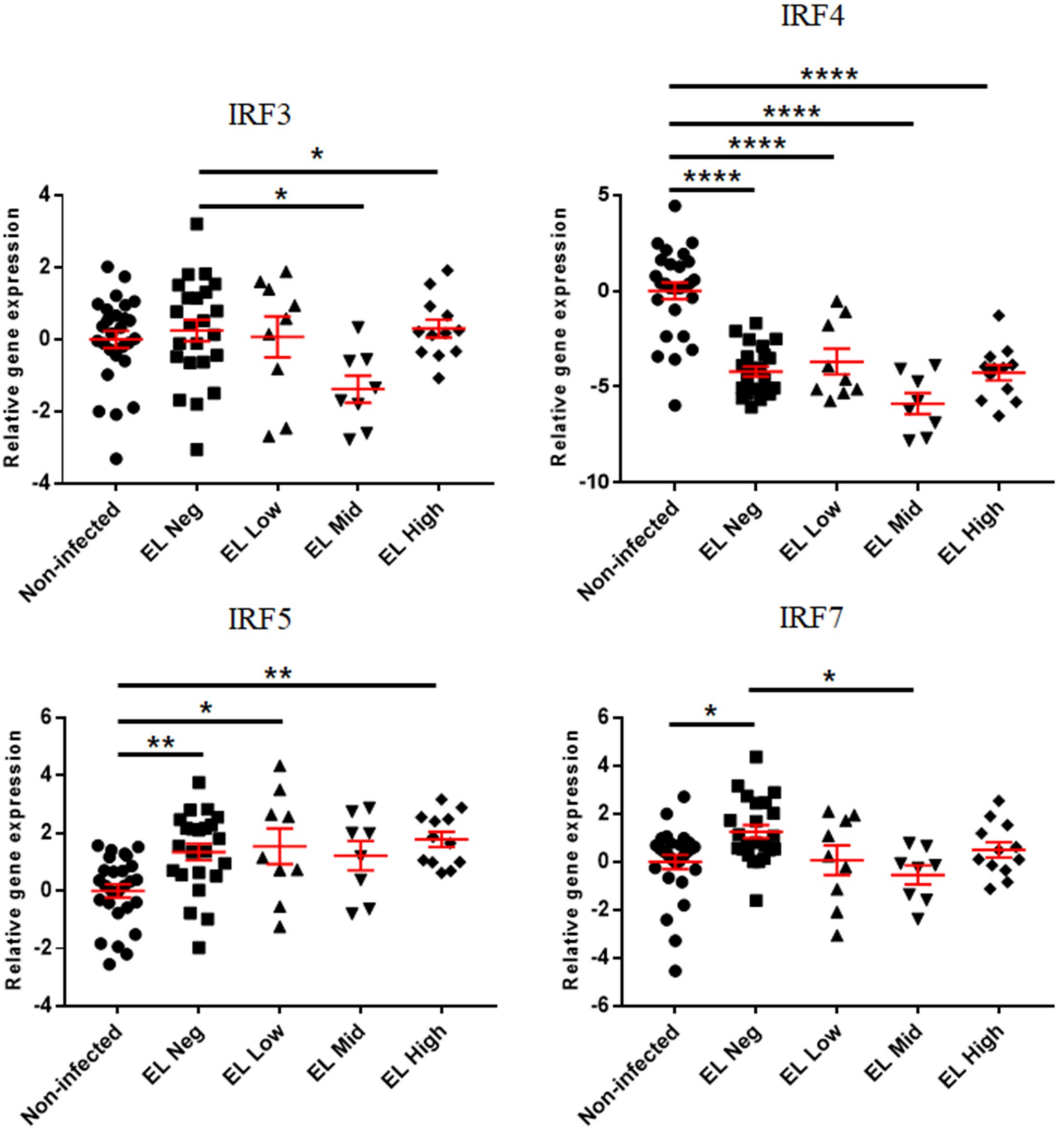
Differences in gene expression levels of interferon regulatory factors between the non-infected, EL Neg, EL Low, EL Mid, and EL High groups. Scatter plots for each gene represent for each individual animal. Values of relative gene expression were normalized to the reference gene β-actin. * indicates a *p*-value <0.05; ** indicates a *p*-value <0.01; *** indicates a *p*-value <0.001; **** indicates a *p*-value <0.0001.

**Fig 5:**
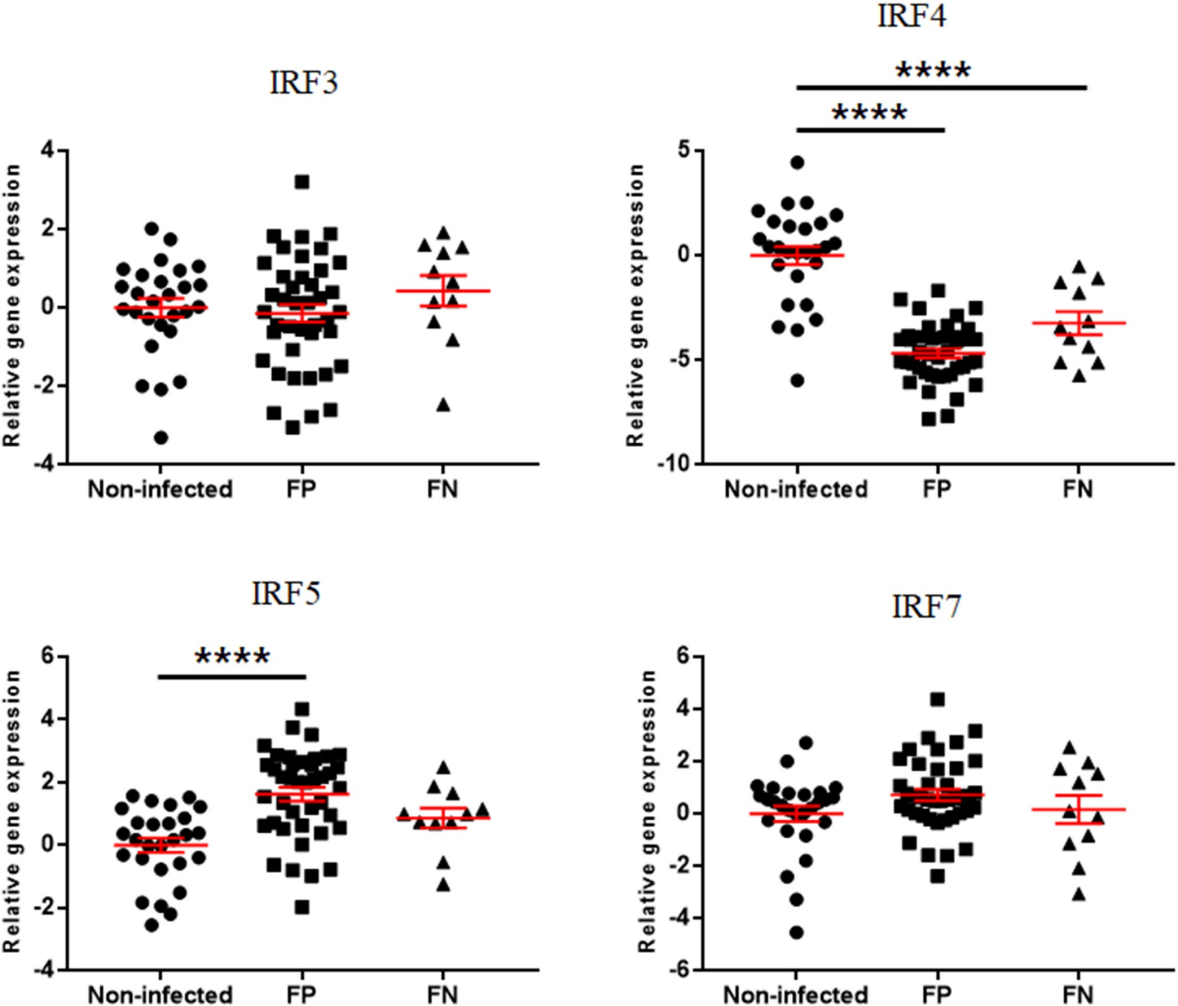
Differences in gene expression level of interferon regulatory factors between non-infected, FP and FN groups. Scatter plots for each gene represent for each individual animal. Values of relative gene expression were normalized to the reference gene β-actin. * indicates a *p*-value <0.05; ** indicates a *p*-value <0.01; *** indicates a *p*-value <0.001; **** indicates a *p*-value <0.0001.

The expressions of interferon regulatory factors are presented in Figs 4 and 5. IRF3 was downregulated in the EL Mid and High groups compared to the EL Neg group. On the other hand, IRF5 was upregulated in the EL Neg, Low, and High groups compared to the non-infected group. The expression of IRF7 was increased in the EL Neg group compared to the non-infected group. In addition, IRF7 was downregulated in the EL Mid group compared to the EL Neg group. According to the fecal shedding-based classification, IRF5 was upregulated in the FP group compared to the non-infected group. In contrast, IRF4 was downregulated in the FP and FN groups compared to the non-infected group. In addition, IRF3 was downregulated in the EL Mid and High groups compared to the EL Neg group.

**Fig 6:**
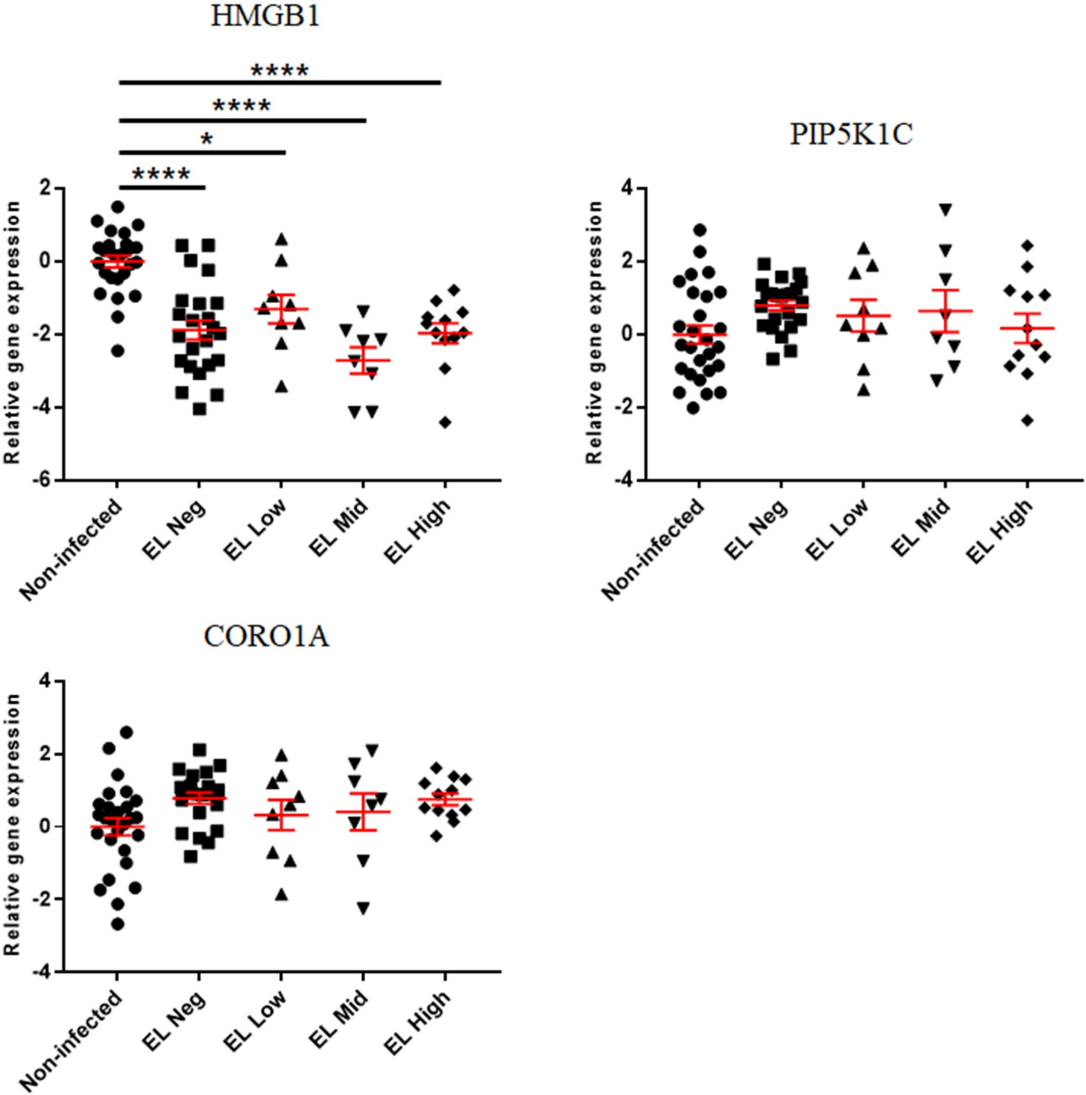
Differences in gene expression level of calcium signaling-associated genes between the non-infected, EL Neg, EL Low, EL Mid, and EL High groups. Scatter plots for each gene represent for each individual animal. Values of relative gene expression were normalized to the reference gene β-actin. * indicates a *p*-value <0.05; ** indicates a *p*-value <0.01; *** indicates a *p*-value <0.001; **** indicates a *p*-value <0.0001.

**Fig 7:**
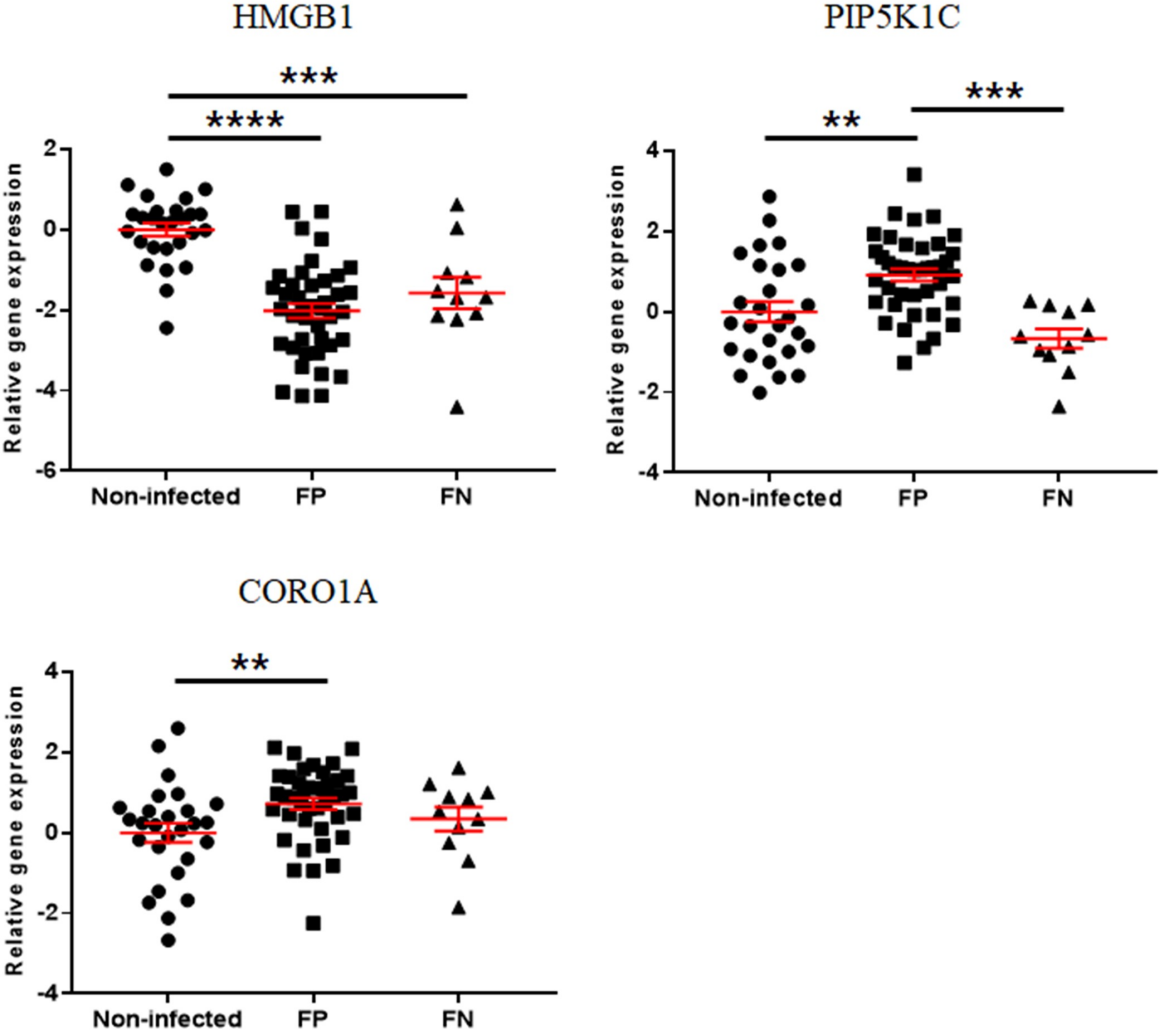
Differences in gene expression levels of calcium signaling-associated genes between the non-infected, FP, and FN groups. Scatter plots for each gene represent for each individual animal. Values of relative gene expression were normalized to the reference gene β-actin. * indicates a *p*-value <0.05; ** indicates a *p*-value <0.01; *** indicates a *p*-value <0.001; **** indicates a *p*-value <0.0001.

The expressions of calcium signaling-associated genes are presented in Figs 6 and 7. HMGB1 was downregulated in the EL Neg, Low, Mid, and High groups compared to the non-infected group. In addition, the expression of HMGB1 was decreased in the FP and FN groups compared to the non-infected group. The expression of PIP5K1C was increased in the FP group compared to the non-infected group and decreased in the FN group compared to the FP group. CORO1A was upregulated only in the FP group compared to the non-infected group. All data of gene expression fold change in this study were recorded in the supplementary materials (S2 and S3 Tables).

## Discussion

Gene expression profiling of whole blood is a useful indicator of the progression of diseases and immune responses in chronic infectious diseases [29, 30]. Although gene expression in whole blood does not completely reflect the immunological changes at the site of infection, alteration of gene expression in peripheral blood may be specific to disease progression and provide useful information for identification of pathophysiology [31–33]. Several studies have investigated the host response to MAP infection in whole blood, peripheral blood mononuclear cells (PBMC), and monocyte-derived macrophages (MDM) [34–40]. However, the host response during the immunological shift period between early subclinical to late subclinical stages were not fully understood. Therefore, the present study was conducted to identify the host response during the subclinical stages that revealed different levels of antibodies and the presence of MAP shedding in feces.

The present study suggests a difference in the gene expression profile of non-infected animals and infected animals with subclinical phases with different levels of fecal shedding and antibodies to MAP. The expression of Th17-derived cytokine genes differed between the experimental groups. Although the expression of all genes was not perfectly matched, Th17-derived cytokine genes were downregulated during the progression of disease. Th17-derived cytokines play an important role in the early stage of mycobacterial infection [41]. IL-26 and IL-17F are the main effector cytokines of the Th17 response and are associated with host defense against intracellular bacteria [42, 43]. IL-26 induces priming of immune cells and direct killing of pathogens via membrane pore formation [44]. Furthermore, previous studies have revealed that IL-26 upregulates tumor necrosis factor (TNF)-related apoptosis-inducing ligand (TRAIL) expression in human NK cells, which induces the elimination of hepatitis C-infected hepatocytes [45]. Moreover, IL-17 provides protective immunity against intracellular pathogens by modulation of Th1 response and neutrophilic recruitment [46, 47]. In addition, Robinson et al. analyzed the expression of Th17 cytokines in tissue samples pooled with jejunum, ileocecal valve, and adjacent lymph node of MAP-infected red deer [48]. Expression of IL-17 was not significantly changed in early stage of infection, but was significantly increased at late stage. On the other hand, the expression of IL-21, IL-22, and IL-23 did not change significantly depending on the stage of infection [48]. In this study, the expression of IL-17A and IL-17F tended to decrease with the progression of disease. This difference may be due to the difference between the host animal (red deer vs cattle) and the sample (jejunum vs whole blood). Park et al. found that the expression of IL-17, IL-22, IL-23 and RORC was upregulated after the stimulation of MAP in PBMC isolated from cattle infected with MAP [49]. This suggests that Th17-derived cytokines play an important role in the early stages of JD. Therefore, downregulation of Th17-derived cytokine genes reflects insufficient immune response for eliminating intracellular MAP during all subclinical stages in this study.

Formation of granuloma is an important factor in the pathogenesis of JD [50]. MAP crosses the intestinal barrier through M cells or enterocytes and are subsequently uptaken by macrophages located in the lamina propria. Thereafter, cytokine production by activated macrophages and MAP antigens attracts new monocytes. Finally, multi-nucleated giant cells and epithelioid cells are formed, resulting in the formation of new granulomas [50]. Formation of granulomas is tightly regulated by immune responses such as TNF-signaling and neutrophil trafficking [51]. Neutrophil recruitment regulated by the TNF/IL-8 axis is essential for the establishment and maintenance of granulomas, which provides protective immunity to the host during mycobacterial infections [51, 52]. Therefore, impaired recruitment of neutrophil can induce unstable maintenance of granulomas. Th17-derived cytokines, including IL-17, IL-23, and IL-26, enhance granuloma integrity through the modulation of neutrophil recruitment via CXCR3 signaling [53]. In addition, PIP5K1C encodes protein phosphatidylinositol phosphate kinases, which regulate E-cadherin sorting for degradation, and increased activity of PIP5K1C induced the downregulation of E-cadherin [54]. A previous study suggested that E-cadherin is expressed in macrophages if the macrophages fail to eliminate intracellular pathogens, which subsequently contributes to the formation of granulomas [55]. Therefore, upregulation of PIP5K1C and downregulation of IL-17A and IL-17F in the whole blood can reflect loss of granuloma integrity, which induces bacterial shedding through feces.

Maturation of phagosomes is an important process in the defense against microbial pathogen [56]. However, intracellular pathogens can survive and replicate in the phagocytes by disrupting phagosome maturation [57]. CORO1A encodes protein coronin 1 in mammalian cells, and is involved in actin dynamics [58]. A recent study revealed that coronin 1 is an essential factor for modulating calcium signaling after the invasion of pathogenic mycobacteria [59]. Coronin 1 modulates physiological Ca^2+^ fluxes and induces the activation of calcineurin, subsequently blocking phagosome–lysosome fusion [59]. Moreover, IL-22 activates phagosome maturation via enhancing calgranulin A expression in MDMs infected with *Mycobacterium tuberculosis*. [60]. Calgranulin A was upregulated in MAP-infected animals and has been proposed as a diagnostic biomarker for subclinical MAP infections [37, 40]. Thus, upregulation of CORO1A and downregulation of IL-22 indicate enhanced intracellular survival of MAP during subclinical stages.

Expression of interferon regulatory genes was different between experimental groups. First, the expression of IRF4 was decreased in all infected groups compared to the non-infected group. IRF4 is an essential factor for the differentiation of T and B cells as well as the generation of plasma cells [61]. In a previous study, IRF4 knockout mice failed to provoke Th1 immune response against *Listeria monocytogenes* infections [62]. Decreased expression of IRF4 can induce downregulation of the Th1 immune response, which can enhance the persistent survival of MAP. HMGB1 expression is upregulated by the IFN-γ that is secreted in activated macrophages [63]. During early infections, mycobacterial pathogen can inhibit the activation of macrophages via the induction of anti-apoptotic and anti-inflammatory response [64, 65]. This result coincided with a previous study that showed downregulation of a complement immune pathway induced by MAP and consequently enhanced intracellular survival in macrophages [38].

Interferon regulatory factor (IRF) is the transcriptional regulator of IFN genes that regulates the immune response to intracellular pathogen. IRF5 and IRF7 have been shown to activate type I interferons including IFN-α, -β, -ω, -ε, -κ, and pro-inflammatory cytokines [17, 66]. IFN-α/β is the most widely expressed type I IFN, which has diverse effects on innate and adaptive immunity [66]. In general, IFN-α/β have been shown to protect the host from intracellular pathogens, including *Chlamydia trachomatis*, *Legionella pneumophila*, and *Salmonella* Typhimurium [67, 68, 69]. More specifically, IFN-α/β inhibit intracellular replication of *Chlamydia trachomatis* through indoleamine 2,3-dioxygenase (IDO) mediated depletion of L-tryptophan [67]. A previous study showed upregulation of IDO in THP-1 monocytes, PBMCs, and intestinal tissues of MAP-infected animals [70]. However, IDO-mediated tryptophan depletion also has a detrimental effect on the host. For example, low concentrations of tryptophan inhibit T cell proliferation [71]. In addition, metabolites of IDO-mediated tryptophan metabolism such as kynurenine, 3-hydroxykynurenine, and 3-hydroxyanthranilic acid can inhibit T cell proliferation via apoptosis and arrest of the cell cycle [72, 73, 74]. Therefore, upregulation of IRF5 and IRF7 can result in the inhibition of T cell proliferation via IDO-mediated tryptophan depletion. In another study, expression of IRF5 was downregulated after 3 weeks of infection with MAP in the spleen of mice [27]. These differences are possibly related to different host species or differences in the first infection time and dose. Taken together, whether IFN-α/β is beneficial or detrimental for the host is not clear in MAP infections. Therefore, the specific role of IFN-α/β during subclinical stages of JD should be identified in further studies.

Several studies revealed molecular mimicry between MAP protein and host IRF5 protein [75, 76, 77]. Mameli et al. identified that molecular mimicry between MAP_4027_18−32_ and IRF5_424-434_ peptide [75]. Also, they found that antibodies to both MAP_4027_18−32_ and IRF5_424-434_ peptides were significantly elevated in sera and cerebrospinal fluid of multiple sclerosis patients when compared to healthy individuals [75]. Cossu et al. found similar result in sera of multiple sclerosis patients and these two peptides induce activation of the Th1 response in the whole blood while suppressing the Th2 response [76]. Recently, Bo et al. analyzed the serum of rheumatoid arthritis patients and suggests exposure to MAP can trigger specific humoral immune response against host IRF5 protein due to molecular mimicry between MAP_4027_18−32_ and IRF5_424-434_ peptides in rheumatoid arthritis patients [77]. Taken together, humoral immune response to IRF5_424-434_ peptide which induced by exposure to MAP may weaken the Th1 response and activate the Th2 response during subclinical stage of JD.

**Fig 8:**
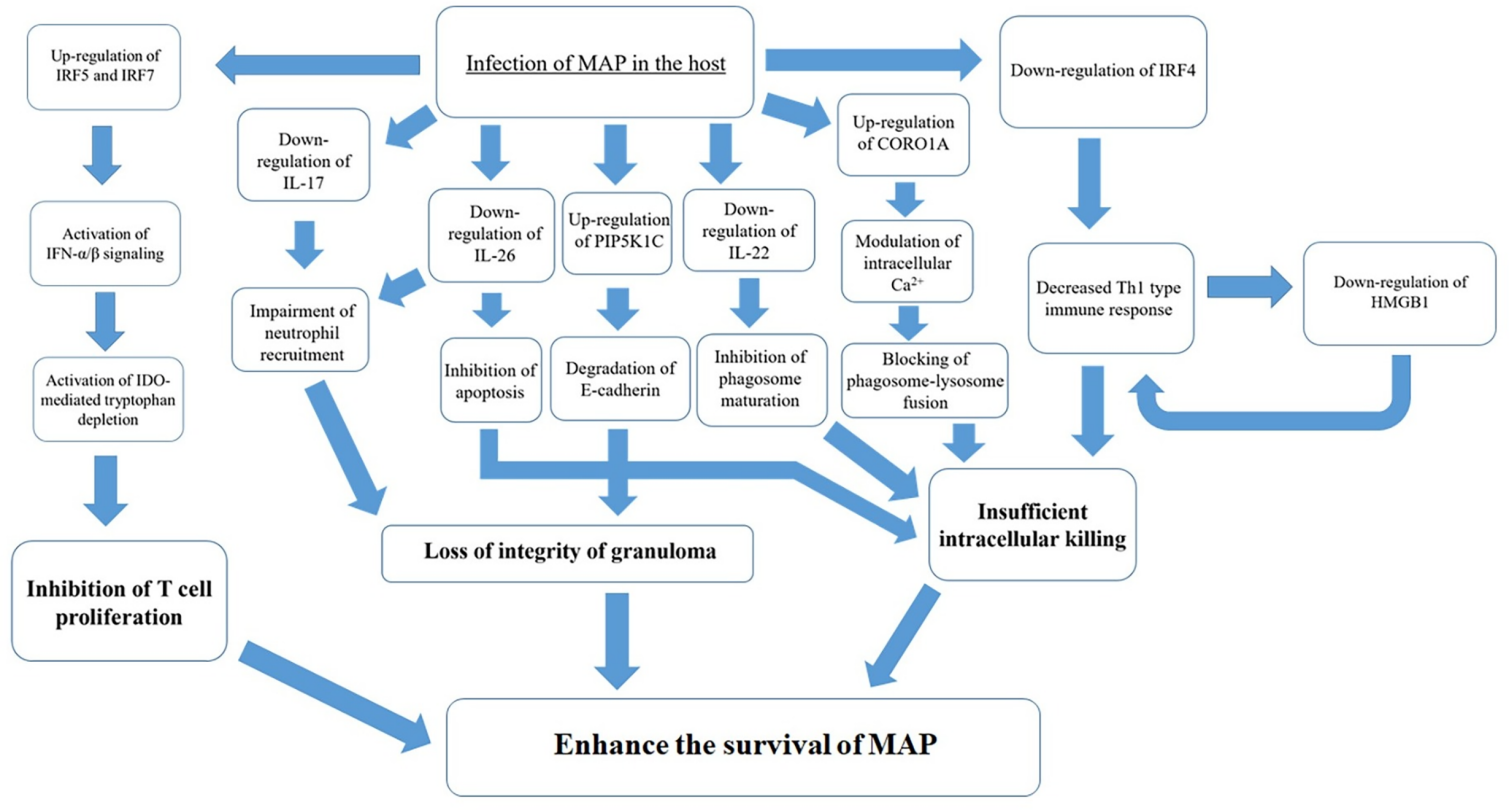
Novel model for the manipulation of host responses by *Mycobacterium avium* subsp. *paratuberculosis* (MAP) for its survival during subclinical stages of Johne’s disease. MAP can manipulate host responses to enhance its survival. Upregulation of PIP5K1C and downregulation of IL-17A, IL-17F, and IL-26 induces a loss of granuloma integrity, which can result in fecal shedding and dissemination of MAP. In addition, downregulation of IRF4 and HMGB1 can impair intracellular elimination of MAP. Upregulation of CORO1A modulates intracellular Ca^2+^, which can block phagosome–lysosome fusion. Moreover, decreased expression of IL-22 indicates an inhibition of phagosome maturation. Upregulation of IRF5 and IRF7 activates IFN-α/β signaling, which upregulate IDO-mediated tryptophan depletion and subsequently induce the inhibition of T cell proliferation. Taken together, a manipulated host response enhances the survival of MAP during the subclinical phases of JD.

In conclusion, we propose a novel model for the host response, which enhances the survival of MAP (Fig 8). Downregulation of IL-17A, IL-17F, IL-26, and upregulation of PIP5K1C and loss of granuloma integrity results in fecal shedding and dissemination of the pathogen. Downregulation of IRF4 resulted in impaired Th1 immune response, which decreased expression of HMGB1 and enhanced the downregulation of the Th1 immune response. In addition, increased expression of IRF5 and IRF7 suggest that activation of IFN-α/β signaling during subclinical stages induce IDO-mediated tryptophan metabolism. IDO-mediated depletion of tryptophan indicates an inhibition of T cell proliferation, subsequently leading to an immunosuppressive state. Upregulation of CORO1A suggest the possibility of the failure to intracellularly eliminate MAP. Taken together, this model suggests manipulation of host responses for the survival of MAP that occurs during the subclinical phases of JD. However, this model was established based on the gene expressions of whole blood, which is not specific to individual immune cell subsets. Therefore, more specific roles of the immune regulatory genes during subclinical phases should be identified via interactions between different immune cells in co-culture systems or *in vivo* in further studies.

## Supporting information

S1 Table Basic characteristics of study subjects. (DOCX)

S2 Table Individual fold change of immune regulatory genes between groups classified based on ELISA S/P ratio. (DOCX)

S3 Table Individual fold change of immune regulatory genes between groups classified based on fecal shedding. (DOCX)
