# Supplementary material for: Gene expression profiles of immune-regulatory genes in whole blood of cattle with a subclinical infection of Mycobacterium avium subsp. paratuberculosis
Source: PLoS One. 2018 Apr 26;13(4):e0196502. doi: 10.1371/journal.pone.0196502 (PMC5919679; doi:10.1371/journal.pone.0196502)
Supplement: S1 Table — (DOCX) [file pone.0196502.s001.docx]

**S1 Table. Basic characteristics of study subjects**

| **Numbers of subjects** | **Non-infected (n=27)** | | **EL neg (n=23)** | | **EL Low (n=9)** | | **EL Mid (n=8)** | | **EL high (n=12)** | | **FP (n=41)** | | **FN (n=11)** | |
| --- | --- | --- | --- | --- | --- | --- | --- | --- | --- | --- | --- | --- | --- | --- |
| Heifers, n (%) | 27 (100) | | 23 (100) | | 9 (100) | | 8 (100) | | 12 (100) | | 41 (100) | | 11 (100) | |
| Median age (years) | 4 (3 to 10) | | 5 (2 to 8) | | 7 (3 to 7) | | 4.5 (4 to 8) | | 6 (4 to 6) | | 5 (2 to 8) | | 6 (3 to 7) | |
| Breed | Holstein | | Holstein | | Holstein | | Holstein | | Holstein | | Holstein | | Holstein | |
|  | Animal No. | Age (years) | Animal No. | Age (years) | Animal No. | Age (years) | Animal No. | Age (years) | Animal No. | Age (years) | Animal No. | Age  (years) | Animal No. | Age (years) |
|  | A01 | 5 | B01 | 7 | C01 | 5 | D01 | 4 | E01 | 6 | B01 | 7 | C02 | 3 |
|  | A02 | 10 | B02 | 5 | C02 | 3 | D02 | 5 | E02 | 4 | B02 | 5 | C03 | 7 |
|  | A03 | 10 | B03 | 8 | C03 | 7 | D03 | 4 | E03 | 4 | B03 | 8 | C04 | 5 |
|  | A04 | 8 | B04 | 7 | C04 | 5 | D04 | 5 | E04 | 6 | B04 | 7 | C05 | 7 |
|  | A05 | 8 | B05 | 7 | C05 | 7 | D05 | 4 | E05 | 4 | B05 | 7 | C07 | 7 |
|  | A06 | 6 | B06 | 4 | C06 | 7 | D06 | 8 | E06 | 6 | B06 | 4 | E07 | 6 |
|  | A07 | 6 | B07 | 5 | C07 | 7 | D07 | 4 | E07 | 6 | B07 | 5 | E08 | 6 |
|  | A08 | 4 | B08 | 3 | C08 | 7 | D08 | 5 | E08 | 6 | B08 | 3 | E09 | 4 |
|  | A09 | 4 | B09 | 5 | C09 | 7 |  | | E09 | 4 | B09 | 5 | E10 | 6 |
|  | A10 | 4 | B10 | 3 |  | |  | | E10 | 6 | B10 | 3 | E11 | 4 |
|  | A11 | 3 | B11 | 3 |  | |  | | E11 | 4 | B11 | 3 | E12 | 4 |
|  | A12 | 3 | B12 | 4 |  | |  | | E12 | 4 | B12 | 4 |  | |
|  | A13 | 6 | B13 | 3 |  | |  | |  | | B13 | 3 |  | |
|  | A14 | 6 | B14 | 3 |  | |  | |  | | B14 | 3 |  | |
|  | A15 | 5 | B15 | 5 |  | |  | |  | | B15 | 5 |  | |
|  | A16 | 5 | B16 | 3 |  | |  | |  | | B16 | 3 |  | |
|  | A17 | 6 | B17 | 5 |  | |  | |  | | B17 | 5 |  | |
|  | A18 | 4 | B18 | 7 |  | |  | |  | | B18 | 7 |  | |
|  | A19 | 3 | B19 | 4 |  | |  | |  | | B19 | 4 |  | |
|  | A20 | 5 | B20 | 5 |  | |  | |  | | B20 | 5 |  | |
|  | A21 | 4 | B21 | 7 |  | |  | |  | | B21 | 7 |  | |
|  | A22 | 3 | B22 | 2 |  | |  | |  | | B22 | 2 |  | |
|  | A23 | 3 | B23 | 3 |  | |  | |  | | B23 | 3 |  | |
|  | A24 | 3 |  |  |  | |  | |  | | C01 | 5 |  | |
|  | A25 | 3 |  |  |  | |  | |  | | C06 | 7 |  | |
|  | A26 | 3 |  |  |  | |  | |  | | C08 | 7 |  | |
|  | A27 | 3 |  |  |  | |  | |  | | C09 | 7 |  | |
|  |  | |  | |  | |  | |  | | D01 | 4 |  | |
|  |  | |  | |  | |  | |  | | D02 | 5 |  | |
|  |  | |  | |  | |  | |  | | D03 | 4 |  | |
|  |  | |  | |  | |  | |  | | D04 | 5 |  | |
|  |  | |  | |  | |  | |  | | D05 | 4 |  | |
|  |  | |  | |  | |  | |  | | D06 | 8 |  | |
|  |  | |  | |  | |  | |  | | D07 | 4 |  | |
|  |  | |  | |  | |  | |  | | D08 | 5 |  | |
|  |  | |  | |  | |  | |  | | E01 | 6 |  | |
|  |  | |  | |  | |  | |  | | E02 | 4 |  | |
|  |  | |  | |  | |  | |  | | E03 | 4 |  | |
|  |  | |  | |  | |  | |  | | E04 | 6 |  | |
|  |  | |  | |  | |  | |  | | E05 | 4 |  | |
|  |  | |  | |  | |  | |  | | E06 | 6 |  | |
