# Supplementary material for: Gene expression profiles of immune-regulatory genes in whole blood of cattle with a subclinical infection of Mycobacterium avium subsp. paratuberculosis
Source: PLoS One. 2018 Apr 26;13(4):e0196502. doi: 10.1371/journal.pone.0196502 (PMC5919679; doi:10.1371/journal.pone.0196502)
Supplement: S2 Table — (DOCX) [file pone.0196502.s002.docx]

**S2 Table. Individual fold change of immune regulatory genes between groups classified based on ELISA S/P ratio.**

| **IRF3** | | | | |
| --- | --- | --- | --- | --- |
| **Non-infected** | **EL neg** | **EL Low** | **EL Mid** | **EL high** |
| 0.832962963 | 1.826296 | 1.39963 | -0.60037 | 0.126296 |
| 1.742962963 | 1.152963 | 0.14963 | -0.55704 | -0.44037 |
| -2.08037037 | 1.312963 | 1.606296 | -1.7937 | -0.3287 |
| -0.277037037 | 0.78963 | -2.45704 | -1.3437 | -1.06704 |
| 0.53962963 | -0.62037 | -0.81704 | -2.7787 | 0.216296 |
| -0.037037037 | 0.132963 | 1.882963 | -1.6937 | 0.252963 |
| -0.973703704 | 1.802963 | -2.6837 | -2.60037 | 1.546296 |
| 0.576296296 | 1.512963 | 0.586296 | 0.332963 | 1.916296 |
| 0.366296296 | -1.4887 | 0.952963 |  | 0.922963 |
| -0.593703704 | -1.68037 |  |  | 0.662963 |
| 0.332962963 | -0.43704 |  |  | -0.34704 |
| 1.216296296 | -1.79704 |  |  | 0.176296 |
| 0.166296296 | -0.4737 |  |  |  |
| 0.94962963 | -0.10704 |  |  |  |
| -0.11037037 | 0.76963 |  |  |  |
| 0.522962963 | 0.516296 |  |  |  |
| -0.097037037 | 0.396296 |  |  |  |
| -0.437037037 | 3.20963 |  |  |  |
| 0.026296296 | -0.11037 |  |  |  |
| 1.05962963 | 1.546296 |  |  |  |
| 0.982962963 | 1.146296 |  |  |  |
| 2.016296296 | -0.65037 |  |  |  |
| 0.666296296 | -3.05037 |  |  |  |
| -0.20037037 |  |  |  |  |
| -1.993703704 |  |  |  |  |
| -1.89037037 |  |  |  |  |
| -3.307037037 |  |  |  |  |
| **IRF4** | | | | |
| **Non-infected** | **EL neg** | **EL Low** | **EL Mid** | **EL high** |
| 1.267283951 | -4.02272 | -5.13605 | -6.18938 | -4.00272 |
| 1.537283951 | -5.05272 | -5.72938 | -4.06605 | -5.79272 |
| 0.153950617 | -5.10272 | -1.78605 | -7.81605 | -4.02938 |
| -0.982716049 | -5.37938 | -0.53605 | -7.67605 | -3.84605 |
| 0.147283951 | -5.38272 | -4.60272 | -5.76938 | -5.72272 |
| 0.133950617 | -5.68605 | -5.12272 | -3.87938 | -6.51605 |
| 0.220617284 | -4.60272 | -1.07605 | -4.72938 | -3.43272 |
| 0.787283951 | -3.88272 | -3.91272 | -6.88272 | -3.14605 |
| 2.130617284 | -3.50938 | -5.32272 |  | -3.95605 |
| 2.513950617 | -3.34272 |  |  | -5.10605 |
| 0.420617284 | -2.87605 |  |  | -4.36272 |
| 1.930617284 | -4.59938 |  |  | -1.28272 |
| -0.342716049 | -2.52938 |  |  |  |
| 2.480617284 | -2.50272 |  |  |  |
| 0.383950617 | -5.58938 |  |  |  |
| 1.617283951 | -5.05938 |  |  |  |
| 0.397283951 | -5.12272 |  |  |  |
| 1.397283951 | -1.67605 |  |  |  |
| 0.587283951 | -5.16605 |  |  |  |
| 4.440617284 | -4.01938 |  |  |  |
| -0.446049383 | -3.42605 |  |  |  |
| -2.369382716 | -2.09272 |  |  |  |
| -3.072716049 | -6.06938 |  |  |  |
| -2.366049383 |  |  |  |  |
| -3.422716049 |  |  |  |  |
| -3.572716049 |  |  |  |  |
| -5.972716049 |  |  |  |  |
| **IRF5** | | | | |
| **Non-infected** | **EL neg** | **EL Low** | **EL Mid** | **EL high** |
| 0.687654321 | 3.750988 | 0.740988 | 0.380988 | 1.064321 |
| 0.860987654 | 2.170988 | 0.727654 | 1.994321 | 0.624321 |
| -1.935679012 | 2.800988 | 1.157654 | -0.79235 | 3.169321 |
| -0.312345679 | 2.167654 | -1.23901 | -0.62568 | 2.410988 |
| -0.019012346 | 2.467654 | 3.510988 | 2.004321 | 2.890988 |
| -0.019012346 | 2.830988 | -0.54235 | 2.749321 | 2.557654 |
| 0.360987654 | 2.107654 | 2.644321 | 1.190988 | 1.874321 |
| 1.290987654 | 2.287654 | 2.567654 | 2.870988 | 0.994321 |
| 1.567654321 | 0.019321 | 4.340988 |  | 1.650988 |
| 0.330987654 | -0.97568 |  |  | 0.690988 |
| 0.167654321 | 1.360988 |  |  | 2.484321 |
| -0.769012346 | 0.957654 |  |  | 0.990988 |
| -0.579012346 | 1.547654 |  |  |  |
| 1.524320988 | -1.97235 |  |  |  |
| -0.419012346 | 0.520988 |  |  |  |
| -1.515679012 | 0.700988 |  |  |  |
| 0.660987654 | 1.347654 |  |  |  |
| 0.710987654 | 2.137654 |  |  |  |
| -0.392345679 | 0.630988 |  |  |  |
| 1.167654321 | 1.814321 |  |  |  |
| 1.220987654 | 2.550988 |  |  |  |
| 1.414320988 | 0.550988 |  |  |  |
| 0.170987654 | -0.77235 |  |  |  |
| 0.387654321 |  |  |  |  |
| -1.829012346 |  |  |  |  |
| -2.192345679 |  |  |  |  |
| -2.542345679 |  |  |  |  |
| **IRF7** | | | | |
| **Non-infected** | **EL neg** | **EL Low** | **EL Mid** | **EL high** |
| 0.802592593 | 1.695926 | 1.952593 | -0.24074 | 0.495926 |
| 2.005925926 | 0.699259 | -1.13407 | 0.772593 | -0.34741 |
| -3.274074074 | 0.805926 | 2.105926 | -1.58741 | 1.900926 |
| -0.014074074 | 0.539259 | -3.05407 | -1.35741 | 0.622593 |
| 0.555925926 | 0.015926 | -2.07407 | -2.37407 | -1.11407 |
| 0.425925926 | 0.495926 | -0.20074 | 0.655926 | 0.112593 |
| -0.247407407 | 2.025926 | 1.099259 | -0.13741 | 1.535926 |
| 0.725925926 | 2.742593 | 0.245926 | -0.06407 | 2.549259 |
| 0.625925926 | 0.145926 | 1.722593 |  | 1.199259 |
| -0.310740741 | 1.735926 |  |  | -0.13407 |
| -0.830740741 | 2.895926 |  |  | 0.109259 |
| 0.782592593 | 0.572593 |  |  | -0.83407 |
| 0.215925926 | 2.455926 |  |  |  |
| 0.695925926 | 1.072593 |  |  |  |
| -0.650740741 | 0.685926 |  |  |  |
| 0.992592593 | 0.282593 |  |  |  |
| 0.025925926 | 0.019259 |  |  |  |
| 0.135925926 | 4.375926 |  |  |  |
| 0.445925926 | 0.549259 |  |  |  |
| 0.545925926 | 3.169259 |  |  |  |
| 0.989259259 | 2.465926 |  |  |  |
| 2.712592593 | 1.132593 |  |  |  |
| 0.312592593 | -1.60241 |  |  |  |
| 1.069259259 |  |  |  |  |
| -2.404074074 |  |  |  |  |
| -1.797407407 |  |  |  |  |
| -4.537407407 |  |  |  |  |
| **IL17A** | | | | |
| **Non-infected** | **EL neg** | **EL Low** | **EL Mid** | **EL high** |
| 0.688271605 | 0.538272 | -0.4084 | -9.8984 | -8.5784 |
| 1.708271605 | -0.43173 | -1.59506 | -8.73506 | -8.73173 |
| -0.265061728 | -1.5184 | 0.598272 | -9.15506 | -5.08333 |
| -0.408395062 | -1.2984 | 0.881605 | -9.21173 | -5.22333 |
| -1.355061728 | -0.69173 | 1.041605 | -5.36 | -6.92333 |
| -0.035061728 | 0.238272 | -6.01667 | -5.06 | -7.14667 |
| -1.065061728 | 0.664938 | -6.10333 | -6.30667 | -0.8884 |
| 0.891604938 | 0.191605 | -5.60667 | -7.55333 | -1.4384 |
| 0.734938272 | -0.0384 | -5.74 |  | -1.1484 |
| 0.238271605 | 0.718272 |  |  | -1.6384 |
| 0.111604938 | 0.858272 |  |  | -1.0584 |
| 0.344938272 | 0.358272 |  |  | -0.86173 |
| -1.145061728 | 2.011605 |  |  |  |
| 1.774938272 | 0.048272 |  |  |  |
| -0.848395062 | -0.67173 |  |  |  |
| 0.138271605 | -0.7984 |  |  |  |
| 0.101604938 | 0.278272 |  |  |  |
| -0.091728395 | 0.788272 |  |  |  |
| 0.011604938 | -0.16173 |  |  |  |
| 1.794938272 | -0.7584 |  |  |  |
| 0.818271605 | 0.188272 |  |  |  |
| 0.464938272 | 1.021605 |  |  |  |
| -0.125061728 | 0.071605 |  |  |  |
| 0.538271605 |  |  |  |  |
| -1.165061728 |  |  |  |  |
| -1.608395062 |  |  |  |  |
| -2.248395062 |  |  |  |  |
| **IL17F** | | | | |
| **Non-infected** | **EL neg** | **EL Low** | **EL Mid** | **EL high** |
| 0.168271605 | -2.9984 | -2.22506 | -4.3184 | -3.76506 |
| 0.541604938 | -2.8384 | -3.96506 | -3.8184 | -3.8484 |
| -0.658395062 | -4.4584 | -0.6484 | -5.88173 | -1.0184 |
| -0.748395062 | -4.8784 | 0.654938 | -4.9184 | -0.53173 |
| -0.608395062 | -2.99506 | 1.318272 | -0.79506 | -4.82506 |
| 0.751604938 | -2.33506 | -3.18173 | -1.0384 | -4.5684 |
| 0.754938272 | -0.79173 | -1.79506 | -1.37173 | -3.60173 |
| 0.124938272 | -1.96173 | -4.62506 | -4.39506 | -5.16506 |
| 2.328271605 | -1.1884 | -3.4084 |  | -4.65506 |
| 1.728271605 | -0.31506 |  |  | -4.48173 |
| -1.135061728 | -0.21506 |  |  | -2.6684 |
| 0.488271605 | -1.1184 |  |  | -1.4884 |
| -2.185061728 | -0.6684 |  |  |  |
| 0.401604938 | -3.44173 |  |  |  |
| -2.841728395 | -3.81173 |  |  |  |
| -0.865061728 | -2.11173 |  |  |  |
| -0.135061728 | -2.01506 |  |  |  |
| 1.098271605 | -2.8684 |  |  |  |
| 0.304938272 | -2.53506 |  |  |  |
| 1.178271605 | -3.23173 |  |  |  |
| 1.054938272 | -3.0384 |  |  |  |
| -0.941728395 | 0.151605 |  |  |  |
| 0.474938272 | -0.86173 |  |  |  |
| 0.904938272 |  |  |  |  |
| 0.371604938 |  |  |  |  |
| -0.565061728 |  |  |  |  |
| -1.991728395 |  |  |  |  |
| **IL22** | | | | |
| **Non-infected** | **EL neg** | **EL Low** | **EL Mid** | **EL high** |
| 0.295925926 | -2.83741 | 5.185926 | -4.67074 | -5.22074 |
| 0.952592593 | -1.38741 | -0.04074 | -4.25074 | -4.24074 |
| -0.274074074 | 0.622593 | 0.642593 | -1.66074 | -1.40741 |
| -1.054074074 | -4.71407 | 2.135926 | -1.79074 | -0.61741 |
| 0.215925926 | -1.76074 | 1.369259 | -0.58407 | -4.53074 |
| 0.382592593 | -0.78741 | -4.04074 | -1.21407 | -3.38741 |
| -0.410740741 | 0.599259 | -0.55741 | -1.33407 | 0.625926 |
| 1.709259259 | -1.13741 | -3.06741 | -2.14407 | 0.895926 |
| 2.929259259 | 1.632593 | -1.89407 |  | -0.79407 |
| 2.425925926 | 3.585926 |  |  | 0.565926 |
| 0.352592593 | 3.165926 |  |  | 2.525926 |
| 1.322592593 | 2.602593 |  |  | -1.87407 |
| -1.607407407 | 3.112593 |  |  |  |
| 1.922592593 | -1.86074 |  |  |  |
| -0.157407407 | -1.84074 |  |  |  |
| 0.385925926 | 0.482593 |  |  |  |
| 0.252592593 | 4.105926 |  |  |  |
| 2.302592593 | 2.945926 |  |  |  |
| 0.135925926 | 3.042593 |  |  |  |
| 3.725925926 | -0.23074 |  |  |  |
| -0.900740741 | 0.255926 |  |  |  |
| -3.314074074 | 5.022593 |  |  |  |
| -0.847407407 | 4.759259 |  |  |  |
| -1.350740741 |  |  |  |  |
| -3.090740741 |  |  |  |  |
| -3.597407407 |  |  |  |  |
| -2.707407407 |  |  |  |  |
| **IL26** | | | | |
| **Non-infected** | **EL neg** | **EL Low** | **EL Mid** | **EL high** |
| 0.787037037 | 1.303704 | -10.033 | -2.90963 | -2.91963 |
| -0.386296296 | 0.25037 | -6.6563 | -2.2663 | -2.97963 |
| 0.533703704 | 0.79037 | 0.073704 | -1.48963 | -1.1763 |
| -0.052962963 | -1.63296 | 1.32037 | -0.62296 | -0.7463 |
| 0.343703704 | 1.163704 | 0.04037 | 1.06037 | -1.01963 |
| -0.53962963 | 1.983704 | -0.2963 | 0.523704 | -1.85963 |
| 2.477037037 | -3.98963 | -0.93296 | 0.42037 | -10.4296 |
| 3.01037037 | -7.01296 | 0.123704 | -2.37963 | -6.45296 |
| 1.79037037 | -4.05296 | -0.0863 |  | -9.84963 |
| 1.423703704 | -5.6063 |  |  | -7.13963 |
| 2.467037037 | -4.6863 |  |  | -3.49963 |
| 0.863703704 | -6.5563 |  |  | -5.11963 |
| -1.47962963 | -4.99963 |  |  |  |
| 0.743703704 | -9.19963 |  |  |  |
| -2.04962963 | -7.3163 |  |  |  |
| -3.092962963 | -5.95963 |  |  |  |
| -1.452962963 | -5.79296 |  |  |  |
| -2.282962963 | -8.52296 |  |  |  |
| -2.932962963 | -10.2563 |  |  |  |
| 1.233703704 | -9.10963 |  |  |  |
| 3.21037037 | -9.0663 |  |  |  |
| -0.956296296 | -6.11296 |  |  |  |
| -2.146296296 | -8.39963 |  |  |  |
| 1.397037037 |  |  |  |  |
| 0.52037037 |  |  |  |  |
| -0.05962963 |  |  |  |  |
| -3.36962963 |  |  |  |  |
| **CORO1A** | | | | |
| **Non-infected** | **EL neg** | **EL Low** | **EL Mid** | **EL high** |
| 2.154320988 | 1.580988 | 1.207654 | 0.574321 | 0.467654 |
| 2.597654321 | 0.877654 | 0.344321 | 0.097654 | 0.324321 |
| -1.455679012 | -0.43901 | 0.837654 | -2.24901 | 1.304321 |
| -0.349012346 | 0.897654 | -1.85235 | -0.94568 | 1.384321 |
| 0.394320988 | -0.18235 | -0.69568 | 1.724321 | 1.194321 |
| 0.540987654 | 0.390988 | 1.974321 | 2.080988 | 0.450988 |
| -2.125679012 | 0.734321 | 0.607654 | 1.234321 | 1.004321 |
| 0.327654321 | 1.107654 | -0.93235 | 0.754321 | 1.614321 |
| 0.624320988 | 0.600988 | 1.410988 |  | 0.524321 |
| 0.190987654 | 1.684321 |  |  | 0.887654 |
| -0.095679012 | 1.410988 |  |  | -0.25235 |
| -0.232345679 | 1.120988 |  |  | 0.140988 |
| 0.234320988 | 1.080988 |  |  |  |
| 0.067654321 | -0.81568 |  |  |  |
| 0.230987654 | 0.994321 |  |  |  |
| -0.179012346 | -0.31568 |  |  |  |
| -0.999012346 | 1.004321 |  |  |  |
| -0.652345679 | 0.900988 |  |  |  |
| 0.257654321 | -0.11901 |  |  |  |
| 0.917654321 | 1.500988 |  |  |  |
| 0.960987654 | 0.940988 |  |  |  |
| 1.427654321 | 2.117654 |  |  |  |
| 0.717654321 | 0.967654 |  |  |  |
| 0.534320988 |  |  |  |  |
| -1.679012346 |  |  |  |  |
| -1.739012346 |  |  |  |  |
| -2.672345679 |  |  |  |  |
| **HMGB1** | | | | |
| **Non-infected** | **EL neg** | **EL Low** | **EL Mid** | **EL high** |
| -0.445061728 | 0.441605 | -2.23173 | -2.14173 | -1.9484 |
| 1.494938272 | -1.07173 | -1.68173 | -1.8984 | -1.61173 |
| -0.475061728 | -1.1584 | 0.624938 | -2.1884 | -2.93173 |
| -0.085061728 | -1.8084 | -1.18173 | -2.7384 | -1.94173 |
| -0.018395062 | -1.44173 | 0.044938 | -4.1284 | -0.78506 |
| 0.378271605 | -1.6184 | -0.94173 | -3.0784 | -1.3884 |
| -1.008395062 | -0.23506 | -3.41173 | -4.13506 | -1.52173 |
| -2.445061728 | -1.56506 | -1.27173 | -1.3784 | -1.0784 |
| 0.298271605 | -3.0684 | -1.68173 |  | -2.0784 |
| -0.885061728 | -4.03506 |  |  | -1.6984 |
| 1.111604938 | -3.6584 |  |  | -2.14506 |
| 1.004938272 | 0.438272 |  |  | -4.40173 |
| 0.181604938 | -2.83506 |  |  |  |
| 0.374938272 | -2.72173 |  |  |  |
| 0.274938272 | -2.3884 |  |  |  |
| -0.045061728 | -1.97173 |  |  |  |
| 0.168271605 | -2.04173 |  |  |  |
| -0.941728395 | -1.14506 |  |  |  |
| 0.444938272 | -2.87506 |  |  |  |
| 0.384938272 | -2.70173 |  |  |  |
| 0.774938272 | -2.1684 |  |  |  |
| 0.471604938 | 0.031605 |  |  |  |
| 0.844938272 | -3.59173 |  |  |  |
| 0.278271605 |  |  |  |  |
| -0.305061728 |  |  |  |  |
| -0.315061728 |  |  |  |  |
| -1.518395062 |  |  |  |  |
| **PIP5K1C** | | | | |
| **Non-infected** | **EL neg** | **EL Low** | **EL Mid** | **EL high** |
| 0.16 | 1.356667 | 0.176667 | -1.26667 | 1.21 |
| 0.52 | 1.063333 | -1.50333 | 0.506667 | 1.08 |
| -1.253333333 | 0.176667 | -0.95 | -0.33 | 1.856667 |
| -0.846666667 | -0.67333 | -0.00667 | -0.09 | 2.436667 |
| -0.526666667 | 0.593333 | 0.266667 | 2.29 | 1.036667 |
| 0.086666667 | 1.113333 | 0.696667 | 3.41 | -0.28333 |
| -0.993333333 | 1.12 | 1.693333 | 1.5 | 0.163333 |
| 0.223333333 | -0.07 | 2.373333 | -0.88667 | -0.56667 |
| -0.343333333 | 1.126667 | 1.9 |  | -2.34667 |
| -0.283333333 | 1.443333 |  |  | -1.07 |
| -1.593333333 | 1.576667 |  |  | -0.61333 |
| 1.46 | 0.426667 |  |  | -0.86333 |
| -1.63 | 1.926667 |  |  |  |
| 1.04 | 1.096667 |  |  |  |
| -2.01 | -0.45 |  |  |  |
| -0.706666667 | 0.413333 |  |  |  |
| -0.926666667 | 0.24 |  |  |  |
| -0.136666667 | 0.883333 |  |  |  |
| -1.586666667 | 0.2 |  |  |  |
| 2.266666667 | 0.78 |  |  |  |
| 2.863333333 | 1.246667 |  |  |  |
| 1.7 | 1.67 |  |  |  |
| 1.16 | 1.036667 |  |  |  |
| 1.15 |  |  |  |  |
| 1.653333333 |  |  |  |  |
| -0.36 |  |  |  |  |
| -1.086666667 |  |  |  |  |
