# Supplementary material for: Gene expression profiles of immune-regulatory genes in whole blood of cattle with a subclinical infection of Mycobacterium avium subsp. paratuberculosis
Source: PLoS One. 2018 Apr 26;13(4):e0196502. doi: 10.1371/journal.pone.0196502 (PMC5919679; doi:10.1371/journal.pone.0196502)
Supplement: S3 Table — (DOCX) [file pone.0196502.s003.docx]

**S3 Table. Individual fold change of immune regulatory genes between groups classified based on fecal shedding.**

|  | **IRF3** |  |
| --- | --- | --- |
| **Non-infected** | **Fecal MAP positive (FP)** | **Fecal MAP negative (FN)** |
| 0.832962963 | -0.60037037 | 1.546296296 |
| 1.742962963 | 0.126296296 | 1.916296296 |
| -2.08037037 | -0.44037037 | 1.39962963 |
| -0.277037037 | -0.55703704 | 0.922962963 |
| 0.53962963 | -1.7937037 | 0.662962963 |
| -0.037037037 | -1.3437037 | -0.347037037 |
| -0.973703704 | 1.882962963 | 0.14962963 |
| 0.576296296 | -0.3287037 | 0.176296296 |
| 0.366296296 | -1.06703704 | 1.606296296 |
| -0.593703704 | -2.7787037 | -2.457037037 |
| 0.332962963 | -1.6937037 | -0.817037037 |
| 1.216296296 | -2.6837037 |  |
| 0.166296296 | -2.60037037 |  |
| 0.94962963 | 0.332962963 |  |
| -0.11037037 | 0.216296296 |  |
| 0.522962963 | 0.252962963 |  |
| -0.097037037 | 0.586296296 |  |
| -0.437037037 | 0.952962963 |  |
| 0.026296296 | 1.826296296 |  |
| 1.05962963 | 1.152962963 |  |
| 0.982962963 | 1.312962963 |  |
| 2.016296296 | 0.78962963 |  |
| 0.666296296 | -0.62037037 |  |
| -0.20037037 | 0.132962963 |  |
| -1.993703704 | 1.802962963 |  |
| -1.89037037 | 1.512962963 |  |
| -3.307037037 | -1.4887037 |  |
|  | -1.68037037 |  |
|  | -0.43703704 |  |
|  | -1.79703704 |  |
|  | -0.4737037 |  |
|  | -0.10703704 |  |
|  | 0.76962963 |  |
|  | 0.516296296 |  |
|  | 0.396296296 |  |
|  | 3.20962963 |  |
|  | -0.11037037 |  |
|  | 1.546296296 |  |
|  | 1.146296296 |  |
|  | -0.65037037 |  |
|  | -3.05037037 |  |
|  | **IRF4** |  |
| **Non-infected** | **Fecal MAP positive (FP)** | **Fecal MAP negative (FN)** |
| 1.267284 | -6.18938272 | -3.432716049 |
| 1.537284 | -4.00271605 | -3.146049383 |
| 0.153951 | -5.79271605 | -5.136049383 |
| -0.98272 | -4.06604938 | -3.956049383 |
| 0.147284 | -7.81604938 | -5.106049383 |
| 0.133951 | -7.67604938 | -4.362716049 |
| 0.220617 | -3.96938272 | -5.729382716 |
| 0.787284 | -4.02938272 | -1.282716049 |
| 2.130617 | -3.84604938 | -1.786049383 |
| 2.513951 | -5.76938272 | -0.536049383 |
| 0.420617 | -3.87938272 | -1.076049383 |
| 1.930617 | -5.32271605 |  |
| -0.34272 | -4.72938272 |  |
| 2.480617 | -6.88271605 |  |
| 0.383951 | -5.72271605 |  |
| 1.617284 | -6.51604938 |  |
| 0.397284 | -4.87938272 |  |
| 1.397284 | -3.91271605 |  |
| 0.587284 | -4.02271605 |  |
| 4.440617 | -5.05271605 |  |
| -0.44605 | -5.10271605 |  |
| -2.36938 | -5.37938272 |  |
| -3.07272 | -5.38271605 |  |
| -2.36605 | -5.68604938 |  |
| -3.42272 | -4.60271605 |  |
| -3.57272 | -3.88271605 |  |
| -5.97272 | -3.50938272 |  |
|  | -3.34271605 |  |
|  | -2.87604938 |  |
|  | -4.59938272 |  |
|  | -2.52938272 |  |
|  | -2.50271605 |  |
|  | -5.58938272 |  |
|  | -5.05938272 |  |
|  | -5.12271605 |  |
|  | -1.67604938 |  |
|  | -5.16604938 |  |
|  | -4.01938272 |  |
|  | -3.42604938 |  |
|  | -2.09271605 |  |
|  | -6.06938272 |  |
|  | **IRF5** |  |
| **Non-infected** | **Fecal MAP positive (FP)** | **Fecal MAP negative (FN)** |
| 0.687654 | 0.38098765 | 1.874320988 |
| 0.860988 | 1.06432099 | 0.994320988 |
| -1.93568 | 0.62432099 | 0.740987654 |
| -0.31235 | 1.99432099 | 1.650987654 |
| -0.01901 | -0.7923457 | 0.690987654 |
| -0.01901 | -0.625679 | 2.484320988 |
| 0.360988 | 3.51098765 | 0.727654321 |
| 1.290988 | 3.16932099 | 0.990987654 |
| 1.567654 | 2.41098765 | 1.157654321 |
| 0.330988 | 2.00432099 | -1.239012346 |
| 0.167654 | 2.74932099 | -0.542345679 |
| -0.76901 | 2.56765432 |  |
| -0.57901 | 1.19098765 |  |
| 1.524321 | 2.87098765 |  |
| -0.41901 | 2.89098765 |  |
| -1.51568 | 2.55765432 |  |
| 0.660988 | 2.64432099 |  |
| 0.710988 | 4.34098765 |  |
| -0.39235 | 3.75098765 |  |
| 1.167654 | 2.17098765 |  |
| 1.220988 | 2.80098765 |  |
| 1.414321 | 2.16765432 |  |
| 0.170988 | 2.46765432 |  |
| 0.387654 | 2.83098765 |  |
| -1.82901 | 2.10765432 |  |
| -2.19235 | 2.28765432 |  |
| -2.54235 | 0.01932099 |  |
|  | -0.975679 |  |
|  | 1.36098765 |  |
|  | 0.95765432 |  |
|  | 1.54765432 |  |
|  | -1.9723457 |  |
|  | 0.52098765 |  |
|  | 0.70098765 |  |
|  | 1.34765432 |  |
|  | 2.13765432 |  |
|  | 0.63098765 |  |
|  | 1.81432099 |  |
|  | 2.55098765 |  |
|  | 0.55098765 |  |
|  | -0.7723457 |  |
|  | **IRF7** |  |
| **Non-infected** | **Fecal MAP positive (FP)** | **Fecal MAP negative (FN)** |
| 0.802593 | -0.240741 | 1.53592593 |
| 2.005926 | 0.4959259 | 2.54925926 |
| -3.27407 | -0.347407 | 1.95259259 |
| -0.01407 | 0.7725926 | 1.19925926 |
| 0.555926 | -1.587407 | -0.1340741 |
| 0.425926 | -1.357407 | 0.10925926 |
| -0.24741 | 2.1059259 | -1.1340741 |
| 0.725926 | 1.9009259 | -0.8340741 |
| 0.625926 | 0.6225926 | 1.72259259 |
| -0.31074 | -2.374074 | -3.0540741 |
| -0.83074 | 0.6559259 | -2.0740741 |
| 0.782593 | 1.0992593 |  |
| 0.215926 | -0.137407 |  |
| 0.695926 | -0.064074 |  |
| -0.65074 | -1.114074 |  |
| 0.992593 | 0.1125926 |  |
| 0.025926 | -0.200741 |  |
| 0.135926 | 0.2459259 |  |
| 0.445926 | 1.6959259 |  |
| 0.545926 | 0.6992593 |  |
| 0.989259 | 0.8059259 |  |
| 2.712593 | 0.5392593 |  |
| 0.312593 | 0.0159259 |  |
| 1.069259 | 0.4959259 |  |
| -2.40407 | 2.0259259 |  |
| -1.79741 | 2.7425926 |  |
| -4.53741 | 0.1459259 |  |
|  | 1.7359259 |  |
|  | 2.8959259 |  |
|  | 0.5725926 |  |
|  | 2.4559259 |  |
|  | 1.0725926 |  |
|  | 0.6859259 |  |
|  | 0.2825926 |  |
|  | 0.0192593 |  |
|  | 4.3759259 |  |
|  | 0.5492593 |  |
|  | 3.1692593 |  |
|  | 2.4659259 |  |
|  | 1.1325926 |  |
|  | -1.602407 |  |
|  | **IL17A** |  |
| **Non-infected** | **Fecal MAP positive (FP)** | **Fecal MAP negative (FN)** |
| 0.688271605 | -9.89839506 | -0.888395062 |
| 1.708271605 | -8.57839506 | -1.438395062 |
| -0.265061728 | -8.7317284 | -0.408395062 |
| -0.408395062 | -8.73506173 | -1.148395062 |
| -1.355061728 | -9.15506173 | -1.638395062 |
| -0.035061728 | -9.2117284 | -1.058395062 |
| -1.065061728 | -6.01666667 | -1.595061728 |
| 0.891604938 | -5.08333333 | -0.861728395 |
| 0.734938272 | -5.22333333 | 0.598271605 |
| 0.238271605 | -5.36 | 0.881604938 |
| 0.111604938 | -5.06 | 1.041604938 |
| 0.344938272 | -5.74 |  |
| -1.145061728 | -6.30666667 |  |
| 1.774938272 | -7.55333333 |  |
| -0.848395062 | -6.92333333 |  |
| 0.138271605 | -7.14666667 |  |
| 0.101604938 | -6.10333333 |  |
| -0.091728395 | -5.60666667 |  |
| 0.011604938 | 0.538271605 |  |
| 1.794938272 | -0.4317284 |  |
| 0.818271605 | -1.51839506 |  |
| 0.464938272 | -1.29839506 |  |
| -0.125061728 | -0.6917284 |  |
| 0.538271605 | 0.238271605 |  |
| -1.165061728 | 0.664938272 |  |
| -1.608395062 | 0.191604938 |  |
| -2.248395062 | -0.03839506 |  |
|  | 0.718271605 |  |
|  | 0.858271605 |  |
|  | 0.358271605 |  |
|  | 2.011604938 |  |
|  | 0.048271605 |  |
|  | -0.6717284 |  |
|  | -0.79839506 |  |
|  | 0.278271605 |  |
|  | 0.788271605 |  |
|  | -0.1617284 |  |
|  | -0.75839506 |  |
|  | 0.188271605 |  |
|  | 1.021604938 |  |
|  | 0.071604938 |  |
|  | **IL17F** |  |
| **Non-infected** | **Fecal MAP positive (FP)** | **Fecal MAP negative (FN)** |
| 0.168272 | -4.31839506 | -3.601728395 |
| 0.541605 | -3.76506173 | -5.165061728 |
| -0.6584 | -3.84839506 | -2.225061728 |
| -0.7484 | -3.81839506 | -4.655061728 |
| -0.6084 | -5.8817284 | -4.481728395 |
| 0.751605 | -4.91839506 | -2.668395062 |
| 0.754938 | -3.1817284 | -3.965061728 |
| 0.124938 | -1.01839506 | -1.488395062 |
| 2.328272 | -0.5317284 | -0.648395062 |
| 1.728272 | -0.79506173 | 0.654938272 |
| -1.13506 | -1.03839506 | 1.318271605 |
| 0.488272 | -1.79506173 |  |
| -2.18506 | -1.3717284 |  |
| 0.401605 | -4.39506173 |  |
| -2.84173 | -4.82506173 |  |
| -0.86506 | -4.56839506 |  |
| -0.13506 | -4.62506173 |  |
| 1.098272 | -3.40839506 |  |
| 0.304938 | -2.99839506 |  |
| 1.178272 | -2.83839506 |  |
| 1.054938 | -4.45839506 |  |
| -0.94173 | -4.87839506 |  |
| 0.474938 | -2.99506173 |  |
| 0.904938 | -2.33506173 |  |
| 0.371605 | -0.7917284 |  |
| -0.56506 | -1.9617284 |  |
| -1.99173 | -1.18839506 |  |
|  | -0.31506173 |  |
|  | -0.21506173 |  |
|  | -1.11839506 |  |
|  | -0.66839506 |  |
|  | -3.4417284 |  |
|  | -3.8117284 |  |
|  | -2.1117284 |  |
|  | -2.01506173 |  |
|  | -2.86839506 |  |
|  | -2.53506173 |  |
|  | -3.2317284 |  |
|  | -3.03839506 |  |
|  | 0.15160494 |  |
|  | -0.8617284 |  |
|  | **IL22** |  |
| **Non-infected** | **Fecal MAP positive (FP)** | **Fecal MAP negative (FN)** |
| 0.295926 | -4.6707407 | 0.625925926 |
| 0.952593 | -5.2207407 | 0.895925926 |
| -0.27407 | -4.2407407 | 5.185925926 |
| -1.05407 | -4.2507407 | -0.79407407 |
| 0.215926 | -1.6607407 | 0.565925926 |
| 0.382593 | -1.7907407 | 2.525925926 |
| -0.41074 | -4.0407407 | -0.04074074 |
| 1.709259 | -1.4074074 | -1.87407407 |
| 2.929259 | -0.6174074 | 0.642592593 |
| 2.425926 | -0.5840741 | 2.135925926 |
| 0.352593 | -1.2140741 | 1.369259259 |
| 1.322593 | -0.5574074 |  |
| -1.60741 | -1.3340741 |  |
| 1.922593 | -2.1440741 |  |
| -0.15741 | -4.5307407 |  |
| 0.385926 | -3.3874074 |  |
| 0.252593 | -3.0674074 |  |
| 2.302593 | -1.8940741 |  |
| 0.135926 | -2.8374074 |  |
| 3.725926 | -1.3874074 |  |
| -0.90074 | 0.62259259 |  |
| -3.31407 | -4.7140741 |  |
| -0.84741 | -1.7607407 |  |
| -1.35074 | -0.7874074 |  |
| -3.09074 | 0.59925926 |  |
| -3.59741 | -1.1374074 |  |
| -2.70741 | 1.63259259 |  |
|  | 3.58592593 |  |
|  | 3.16592593 |  |
|  | 2.60259259 |  |
|  | 3.11259259 |  |
|  | -1.8607407 |  |
|  | -1.8407407 |  |
|  | 0.48259259 |  |
|  | 4.10592593 |  |
|  | 2.94592593 |  |
|  | 3.04259259 |  |
|  | -0.2307407 |  |
|  | 0.25592593 |  |
|  | 5.02259259 |  |
|  | 4.75925926 |  |
|  | **IL26** |  |
| **Non-infected** | **Fecal MAP positive (FP)** | **Fecal MAP negative (FN)** |
| 0.787037 | -2.9096296 | -10.42963 |
| -0.3863 | -2.9196296 | -6.452963 |
| 0.533704 | -2.9796296 | -10.032963 |
| -0.05296 | -2.2662963 | -9.8496296 |
| 0.343704 | -1.4896296 | -7.1396296 |
| -0.53963 | -0.622963 | -3.4996296 |
| 2.477037 | -0.2962963 | -6.6562963 |
| 3.01037 | -1.1762963 | -5.1196296 |
| 1.79037 | -0.7462963 | 0.0737037 |
| 1.423704 | 1.0603704 | 1.32037037 |
| 2.467037 | 0.5237037 | 0.04037037 |
| 0.863704 | -0.0862963 |  |
| -1.47963 | 0.4203704 |  |
| 0.743704 | -2.3796296 |  |
| -2.04963 | -1.0196296 |  |
| -3.09296 | -1.8596296 |  |
| -1.45296 | -0.932963 |  |
| -2.28296 | 0.1237037 |  |
| -2.93296 | 1.3037037 |  |
| 1.233704 | 0.2503704 |  |
| 3.21037 | 0.7903704 |  |
| -0.9563 | -1.632963 |  |
| -2.1463 | 1.1637037 |  |
| 1.397037 | 1.9837037 |  |
| 0.52037 | -3.9896296 |  |
| -0.05963 | -7.012963 |  |
| -3.36963 | -4.052963 |  |
|  | -5.6062963 |  |
|  | -4.6862963 |  |
|  | -6.5562963 |  |
|  | -4.9996296 |  |
|  | -9.1996296 |  |
|  | -7.3162963 |  |
|  | -5.9596296 |  |
|  | -5.792963 |  |
|  | -8.522963 |  |
|  | -10.256296 |  |
|  | -9.1096296 |  |
|  | -9.0662963 |  |
|  | -6.112963 |  |
|  | -8.3996296 |  |
|  | **CORO1A** |  |
| **Non-infected** | **Fecal MAP positive (FP)** | **Fecal MAP negative (FN)** |
| 2.154321 | 0.574321 | 1.00432099 |
| 2.597654 | 0.4676543 | 1.61432099 |
| -1.45568 | 0.324321 | 1.20765432 |
| -0.34901 | 0.0976543 | 0.52432099 |
| 0.394321 | -2.249012 | 0.88765432 |
| 0.540988 | -0.945679 | -0.2523457 |
| -2.12568 | 1.974321 | 0.34432099 |
| 0.327654 | 1.304321 | 0.14098765 |
| 0.624321 | 1.384321 | 0.83765432 |
| 0.190988 | 1.724321 | -1.8523457 |
| -0.09568 | 2.0809877 | -0.695679 |
| -0.23235 | 1.4109877 |  |
| 0.234321 | 1.234321 |  |
| 0.067654 | 0.754321 |  |
| 0.230988 | 1.194321 |  |
| -0.17901 | 0.4509877 |  |
| -0.99901 | 0.6076543 |  |
| -0.65235 | -0.932346 |  |
| 0.257654 | 1.5809877 |  |
| 0.917654 | 0.8776543 |  |
| 0.960988 | -0.439012 |  |
| 1.427654 | 0.8976543 |  |
| 0.717654 | -0.182346 |  |
| 0.534321 | 0.3909877 |  |
| -1.67901 | 0.734321 |  |
| -1.73901 | 1.1076543 |  |
| -2.67235 | 0.6009877 |  |
|  | 1.684321 |  |
|  | 1.4109877 |  |
|  | 1.1209877 |  |
|  | 1.0809877 |  |
|  | -0.815679 |  |
|  | 0.994321 |  |
|  | -0.315679 |  |
|  | 1.004321 |  |
|  | 0.9009877 |  |
|  | -0.119012 |  |
|  | 1.5009877 |  |
|  | 0.9409877 |  |
|  | 2.1176543 |  |
|  | 0.9676543 |  |
|  | **HMGB1** |  |
| **Non-infected** | **Fecal MAP positive (FP)** | **Fecal MAP negative (FN)** |
| -0.44506 | -2.141728395 | -1.521728395 |
| 1.494938 | -1.948395062 | -1.078395062 |
| -0.47506 | -1.611728395 | -2.231728395 |
| -0.08506 | -1.898395062 | -2.078395062 |
| -0.0184 | -2.188395062 | -1.698395062 |
| 0.378272 | -2.738395062 | -2.145061728 |
| -1.0084 | -0.941728395 | -1.681728395 |
| -2.44506 | -2.931728395 | -4.401728395 |
| 0.298272 | -1.941728395 | 0.624938272 |
| -0.88506 | -4.128395062 | -1.181728395 |
| 1.111605 | -3.078395062 | 0.044938272 |
| 1.004938 | -3.411728395 |  |
| 0.181605 | -4.135061728 |  |
| 0.374938 | -1.378395062 |  |
| 0.274938 | -0.785061728 |  |
| -0.04506 | -1.388395062 |  |
| 0.168272 | -1.271728395 |  |
| -0.94173 | -1.681728395 |  |
| 0.444938 | 0.441604938 |  |
| 0.384938 | -1.071728395 |  |
| 0.774938 | -1.158395062 |  |
| 0.471605 | -1.808395062 |  |
| 0.844938 | -1.441728395 |  |
| 0.278272 | -1.618395062 |  |
| -0.30506 | -0.235061728 |  |
| -0.31506 | -1.565061728 |  |
| -1.5184 | -3.068395062 |  |
|  | -4.035061728 |  |
|  | -3.658395062 |  |
|  | 0.438271605 |  |
|  | -2.835061728 |  |
|  | -2.721728395 |  |
|  | -2.388395062 |  |
|  | -1.971728395 |  |
|  | -2.041728395 |  |
|  | -1.145061728 |  |
|  | -2.875061728 |  |
|  | -2.701728395 |  |
|  | -2.168395062 |  |
|  | 0.031604938 |  |
|  | -3.591728395 |  |
|  | **PIP5K1C** |  |
| **Non-infected** | **Fecal MAP positive (FP)** | **Fecal MAP negative (FN)** |
| 0.16 | -1.2666667 | 0.163333333 |
| 0.52 | 1.21 | -0.566666667 |
| -1.25333 | 1.08 | 0.176666667 |
| -0.84667 | 0.50666667 | -2.346666667 |
| -0.52667 | -0.33 | -1.07 |
| 0.086667 | -0.09 | -0.613333333 |
| -0.99333 | 0.69666667 | -1.503333333 |
| 0.223333 | 1.85666667 | -0.863333333 |
| -0.34333 | 2.43666667 | -0.95 |
| -0.28333 | 2.29 | -0.006666667 |
| -1.59333 | 3.41 | 0.266666667 |
| 1.46 | 1.9 |  |
| -1.63 | 1.5 |  |
| 1.04 | -0.8866667 |  |
| -2.01 | 1.03666667 |  |
| -0.70667 | -0.2833333 |  |
| -0.92667 | 1.69333333 |  |
| -0.13667 | 2.37333333 |  |
| -1.58667 | 1.35666667 |  |
| 2.266667 | 1.06333333 |  |
| 2.863333 | 0.17666667 |  |
| 1.7 | -0.6733333 |  |
| 1.16 | 0.59333333 |  |
| 1.15 | 1.11333333 |  |
| 1.653333 | 1.12 |  |
| -0.36 | -0.07 |  |
| -1.08667 | 1.12666667 |  |
|  | 1.44333333 |  |
|  | 1.57666667 |  |
|  | 0.42666667 |  |
|  | 1.92666667 |  |
|  | 1.09666667 |  |
|  | -0.45 |  |
|  | 0.41333333 |  |
|  | 0.24 |  |
|  | 0.88333333 |  |
|  | 0.2 |  |
|  | 0.78 |  |
|  | 1.24666667 |  |
|  | 1.67 |  |
|  | 1.03666667 |  |
